# Exploring the Implementation Potential of Integrating Diabetes Care Into Tuberculosis Services in Pakistan: A Qualitative Case Study

**DOI:** 10.1111/hex.70923

**Published:** 2026-11-09

**Authors:** Saima Afaq, Syeda Fatima Jamal, Aliya Rehman, Urooj Ashfaq, Zala, Muhammad Asim, Farman Ullah Khan, Suneel Gill, Haroon Latif Khan, Mariyam Sarfraz, Zohaib Khan, Kamran Siddiqi, Hannah Jennings

**Affiliations:** ^1^ Institute of Public Health & Social Sciences (IPH&SS) Khyber Medical University Peshawar Pakistan; ^2^ Department of Health Sciences, Heslington University of York York UK; ^3^ Health Services Academy Islamabad Pakistan; ^4^ Hull York Medical School York UK

**Keywords:** CFIR, diabetes, implementation strategies, integrated care, proctor's framework, qualitative research, tuberculosis

## Abstract

**Background:**

The co‐occurrence of diabetes and tuberculosis (TB) worsens outcomes for both conditions, increasing mortality risk and treatment failure. Despite the need for integrated care for the two conditions, evidence on feasible and context‐specific implementation strategies remains limited. This study explored the implementation potential of contextually designed implementation strategies to integrate diabetes within TB care in Pakistan, using the Optimised TB and Diabetes (Opt‐TBD) package.

**Methods:**

This qualitative case study was conducted from October 2023 to June 2024, across four TB facilities representing primary, secondary and tertiary levels of care in Pakistan. Using a qualitative design, we conducted in‐depth interviews with TB patients with comorbid diabetes (*n* = 12) at 3 and 6 months after implementation, while healthcare providers (*n* = 6) were interviewed towards the end of the implementation period. We applied the Consolidated Framework for Implementation Research (CFIR) alongside Proctor's implementation outcomes framework to explore implementation processes and six implementation outcomes: acceptability, appropriateness, feasibility, adoption, penetration and sustainability.

**Results:**

Several contextually tailored implementation strategies, including structured staff training, standardised screening protocols and strengthened referral mechanisms, were employed to integrate diabetes care within routine TB services. Overall, the intervention aligned well with existing TB workflows, provided that adequate resources, clearly defined referral pathways and ongoing staff training were ensured. Despite logistical challenges—such as delays in obtaining HbA1c test results and patient privacy concerns—healthcare providers reported adopting diabetes screening and counselling practices beyond the initial study participants. Strong leadership engagement and consistent programmatic support facilitated early implementation; however, sustaining these efforts long‐term will require securing dedicated financial resources and formal integration into policy frameworks.

**Conclusion:**

This qualitative study indicates promising implementation potential for integrating the Opt‐TBD care package within routine TB services in Pakistan. Participants generally perceived the package as acceptable and workable within routine TB services, while laboratory delays, referral challenges and dependence on external financing remained important barriers to wider implementation. These findings informed refinement of the Opt‐TBD package and the subsequent broader RE‐AIM evaluation.

**Lived Experience or Public Contribution:**

To develop a more inclusive and contextually relevant integrated TB–diabetes care pathway, we gathered input from both service providers and service users, including patients with TB–diabetes multimorbidity. The Opt‐TBD care package was co‐designed with relevant stakeholders, and this qualitative study was undertaken to capture the experiences and insights of patients and healthcare providers during implementation. Participation was sought across different genders, age groups, provinces and levels of care to ensure a broad range of perspectives. This approach helped ensure that the views of those directly affected by and involved in service delivery informed the refinement of the care package and its implementation strategies.

**Abbreviations**
BMUS: basic management units
CFIR: Consolidated Framework for Implementation Research
DTA: district TB assistant
ERIC: Expert Recommendations for Implementing Change
KP: Khyber Pakhtunkhwa
LMICs: low‐ and middle‐income countries
MOs: medical officers
NCDs: non‐communicable diseases
Opt‐TBD: optimised TB and diabetes
RBG: random blood glucose
RE‐AIM: Reach, Effectiveness, Adoption, Implementation and Maintenance
TB: tuberculosis
The Union: The International Union Against Tuberculosis and Lung Disease
ToT: training‐of‐trainers
WHO: World Health Organization

## 1 Introduction

Tuberculosis (TB) remains a major global health challenge, disproportionately affecting low‐ and middle‐income countries, including Pakistan [1]. In 2019, there were approximately 570,000 TB cases in Pakistan and an estimated 44,000 TB‐related deaths, reflecting a substantial disease burden among already vulnerable populations [2]. While established risk factors such as poverty, malnutrition, overcrowding, and HIV/AIDS are well‐recognised, the impact of non‐communicable diseases (NCDs), notably diabetes, on TB outcomes has gained increasing attention [3]. Evidence from systematic reviews suggests that diabetes triples the risk of developing TB and doubles the risk of poor treatment outcomes [4, 5, 6]. A systematic review and meta‐analysis of diabetes among TB patients in South Asia found a pooled prevalence of diabetes in TB patients ranging from 11% to 24% [7]. Bidirectional screening programmes in Pakistan have reported newly diagnosed diabetes in about 24% of TB patients [8].

The interplay between TB and diabetes is multifaceted [9]. Patients with both conditions often face higher treatment failure rates, increased mortality and suboptimal quality of life [9]. In addition to increasing the risk of TB onset, diabetes impairs the immune response, potentially prolonging the infectious period and increasing TB transmission [10]. Anti‐TB therapy itself may exacerbate hyperglycaemia, further complicating the clinical course [11]. Compared to TB patients without diabetes, nearly twice as many patients with comorbid TB–diabetes at 2–3 months show culture positivity [12]. This is attributed to the increased bacterial load of *M. tuberculosis* because of diabetes, resulting in a longer time to culture negativity [9]. Poorly controlled diabetes (as measured by high HbA1c or fasting blood glucose) doubles the risk of mortality during TB treatment as it worsens the TB treatment outcomes [12]. The pooled estimates of a systematic review indicated a higher risk of treatment failure along with a twofold higher risk of mortality in TB–diabetes patients compared to TB patients without diabetes [7]. Furthermore, patients with both TB and diabetes multimorbidity are at increased risk of cardiovascular complications such as myocardial infarction and stroke, resulting in higher mortality rates during the first 2 months of treatment in TB patients with diabetes [13].

Global guidelines and various advocacy groups recommend the integration of TB and diabetes services to improve patient outcomes [14]. However, limited context‐specific evidence and the absence of evidence‐based implementation strategies have hindered effective, tailored solutions in Pakistan [15]. Pakistan's National TB programme lacks structural mechanisms and interdepartmental coordination required for integration of diabetes services into routine TB care. Lack of an allocated budget, a protocol for diabetes screening and diagnosis and the absence of referral pathways limit the integration potential [16]. Despite a robust TB control programme in Pakistan, the management of TB–diabetes comorbidity remains fragmented and inadequately addressed, as currently no protocol is in place for identifying which implementation strategies would enable TB staff to routinely screen, diagnose and refer diabetes cases, nor has the acceptability or feasibility of specific strategies been evaluated [15]. This gap leads to missed opportunities for screening, prevention, timely diagnosis and patient‐centred management [15]. To address this gap, a comprehensive, culturally contextualised and integrated TB–diabetes care package (Opt‐TBD) was co‐designed by the research team and relevant stakeholders [15]. In the current study, we explored the implementation potential of the Opt‐TBD care package and its contextually designed implementation strategies within selected TB care facilities in Pakistan. We use ‘implementation potential’ as an overarching descriptive concept referring to the extent to which the package and its implementation strategies demonstrated promise for integration into routine TB services prior to wider‐scale implementation. It is not treated as a distinct implementation outcome; rather, implementation potential was explored through six established outcomes from Proctor's implementation outcomes framework: acceptability, appropriateness, feasibility, adoption, penetration and sustainability [17]. Similar use of implementation potential to inform implementation planning and wider delivery has been reported in previous implementation research [18]. The Opt‐TBD care package is the clinical aspect of integrated care, and the implementation strategies are employed for its uptake and adoption [17, 19].

This qualitative evaluation was undertaken as an initial step to inform the refinement and contextual optimisation of the Opt‐TBD care package and its associated implementation strategies before their broader implementation and evaluation. A focused assessment of feasibility before broader‐scale implementation evaluation is consistent with best practices in implementation research, ensuring informed refinements and enhanced effectiveness of future interventions [19, 20, 21]. The findings from this study therefore provide formative evidence relevant to the Reach, Effectiveness, Adoption, Implementation and Maintenance (RE‐AIM) [22] dimensions of the subsequent larger‐scale evaluation.

## 2 Materials and Methods

### 2.1 Study Design

We conducted a qualitative case study from October 2023 to June 2024 across four basic management units (BMUs) in Khyber Pakhtunkhwa and Punjab, Pakistan. The case was defined as the implementation of the Opt‐TBD integrated care package and associated implementation strategies within routine TB services across the four purposively selected BMUs. The study was undertaken as a formative qualitative assessment before wider‐scale implementation and evaluation. Patient interviews were conducted at Months 3 and 6 after implementation to examine change and continuity in experiences of receiving integrated TB–diabetes care. Healthcare providers were interviewed towards the end of implementation to explore their experiences of delivering and integrating the package within routine practice. The two patient interview rounds enabled exploration of whether early experiences, barriers and perceptions persisted or changed as implementation progressed.

### 2.2 Study Setting

This study was conducted in BMUs in Pakistan, which are managed by the national and provincial TB control programmes. These BMUs provide diagnosis (including sputum microscopy), registration and treatment for drug‐susceptible TB. A BMU has laboratory staff performing smear microscopy, Xpert MTB/RIF assays and medical staff trained to diagnose and initiate TB treatment [23]. These BMUs operate at all facility levels—primary, secondary and tertiary, with each BMU staffed by trained personnel and equipped with the necessary diagnostic tools and medications. A primary BMU is located at Basic Health Units and Rural Health Centres serving rural and peri‐urban areas, with basic diagnostic capacity; secondary‐level BMUs, based at Tehsil and District Headquarters Hospitals in town and district centres, with a broader diagnostic and referral capacity; and tertiary‐level BMUs, in teaching specialised hospitals in major cities, providing the most comprehensive diagnostic infrastructure and specialist support for complicated cases [24].

Four BMUs were purposively selected in consultation with the respective provincial TB programme managers. Khyber Pakhtunkhwa and Punjab were selected because the feasibility study built on existing collaborations with the provincial TB programmes and aimed to assess implementation across more than one province while remaining feasible within available resources and logistical constraints. Within these provinces, sites were prioritised based on healthcare‐level representation, availability of established TB services and infrastructure, and an average caseload of at least 30 newly diagnosed TB patients per month. Accordingly, two primary‐, one secondary‐, and one tertiary‐level BMU were selected to capture variation in patient volume, staff capacity, and referral pathways. All four selected BMUs proceeded to implementation, and no selected site was subsequently excluded from this study.

### 2.3 Optimised TB and Diabetes (Opt‐TBD) Integrated Care Package

The clinical components of the Opt‐TBD integrated care package included:

1. *Diabetes screening and diagnosis*: Random blood glucose (RBG) testing was conducted for all newly diagnosed (≤ 4 weeks) adult (≥ 18 years) pulmonary TB patients. Patients with RBG ≥ 110 mg/dL or a known history of diabetes underwent HbA1c testing. Confirmed diabetes or prediabetes cases were recorded, and diabetes management plans were integrated into routine TB follow‐up schedules.
2. *Integrated care pathways*: TB patients diagnosed with diabetes received routine TB treatment alongside diabetes monitoring (RBG/HbA1c measurements at baseline, 3 and 6 months) at the BMUs. Patients with uncontrolled or complicated diabetes were referred to specialised care using a newly established referral mechanism.
3. *Counselling and lifestyle modification*: Patients and family members received TB and diabetes counselling sessions at baseline, 3 and 6 months. Counselling covered prediabetes and diabetes education, healthy lifestyle advice and smoking cessation support.

Implementation strategies, as contextually designed methods to support integration, include staff training, enhanced referral mechanisms, modified data recording systems, patient incentives, and strategic programme leadership engagement. Although conceptually distinct, the care package and its implementation strategies are closely interlinked—effective delivery of the care package components inherently depends on the successful deployment of these strategies. Thus, our assessment explored the implementation potential of the Opt‐TBD care package through these contextually tailored strategies, reflecting real‐world integration scenarios. We employed the Consolidated Framework for Implementation Research (CFIR), specifically leveraging its ‘Process domain’ to identify and ensure the inclusion of crucial implementation steps (e.g., engaging stakeholders, executing training, reflecting and evaluating practices). CFIR served as a practical guide for strategy operationalisation, while Proctor's outcomes framework provided the structure for evaluating the resulting implementation potential. Table 1 summarises the operationalisation of implementation strategies aligned with CFIR's Process domain constructs and maps them against Proctor's implementation outcomes. The conceptual relationship between the Opt‐TBD care package, implementation strategies, and implementation outcomes is shown in Figure 1.

**Table 1:** Opt‐TBD implementation strategies mapped to the ERIC strategies and aligned with the CFIR process domain.

ERIC cluster and strategies	CFIR—Process domain constructs			
	Reflecting and Evaluating (Implementation and Innovation)			
	Qualitative data related to the content and implementation of the Opt‐TBD care package will be collected and discussed to assess the success/outcomes of the implementation		
	Engaging	Doing	Milestone	Outcomes affected^a^
1. Support clinicians				
Revise professional roles.	TB programme management and staff.	Meeting with TB programme management and the site staff. Supervision of the diabetes data, in addition to the routine data, collected at BMUs by the TB District Focal Person.	Task sharing and defining the roles of TB staff.	Acceptability Adoption Penetration Appropriateness
2. Develop stakeholder interrelationships				
Promote network weaving.	TB staff at the facility, specialist medical/diabetes care.	Establish an agreement between the BMU and the nearest specialised medical/diabetes care and diagnostic laboratory, transport blood samples, provide specific referral slips to all patients and maintain a referral log.	Addition of referral pathway for diabetes care to the routine TB care.	Acceptability Sustainability Appropriateness Feasibility Penetration
Involve executive boards.	TB programme administration/management.	Include updates on implementing integrated TB and diabetes care in regular TB programme meetings.	Embedding an agenda for integrated TB and diabetes care and relevant data review in routine district, provincial and national‐level TB management meetings.	
3. Train and educate stakeholders				
Develop educational materials	Stakeholder consultations including service providers and service users.	Develop and format manuals, toolkits, and other supporting materials related to integrated TB and diabetes care.	Enable learning about TB and diabetes integrated care for the TB district focal persons and the TB staff.	Acceptability Adoption
Distribute educational materials	TB programme staff	Relevant protocols for screening and diagnosis of diabetes, management algorithm, as well as Opt‐TBD counselling, will be distributed to the TB staff across all study sites at the BMUs.	Improved learning and quality of practice by the TB staff.	Acceptability Adoption
Use train‐the‐trainer strategies.	TB district focal person and TB staff.	ToT workshop for master trainers who will then train the TB staff in each study district. Training on integrated care for diabetes will be added to the routine training of the TB staff.	Integration of Opt‐TBD care within the routine training.	Acceptability Adoption
4. Change Infrastructure				
Change record systems.	TB administration and TB staff at the facility	Inclusion of specific indicators in the existing TB R&R tools (TB01 card and TB03 register): (i) diabetes status (known/unknown); (ii) RBG value at months 0, 3 and 6; (iii) HbA1c levels at month 0, 3 and 6, if RBG is above the threshold; (iv) Referral at month 0, 3 and 6; (v) Counselling at month 0,3, and 6; and (vi) Monthly treatment monitoring.	Updated R & R tools with diabetes indicators provided to all study sites.	Acceptability Feasibility Adoption
Change physical structure and equipment	TB programme administration/management	Provision of testing kits, sampling tubes, syringes and glucometers for diabetes testing to the BMUs.	On‐site availability of the recommended diagnostic tools.	Acceptability Feasibility Adoption
5. Utilise financial strategies				
Use capitated payments	DOTS Strategy Facilitators	Pay the DOTS Strategy Facilitators and TB focal persons/master trainers a set amount per patient (with TB and diabetes multimorbidity cluster) for delivering the Opt‐TBD.	Incentivise the integrated care practice for the TB staff.	Acceptability Adoption
6. Engage consumers				
Involve patients/consumers and family members	Patients visiting the BMU (service users).	Financial incentives (transport allowance) will be provided to the patients who come for their HbA1c results to encourage them to follow up. Patients will be actively engaged in their Tb–diabetes management plan.	Improving patients' compliance with recommended medical tests and follow‐ups.	Acceptability Adoption Feasibility
	Adaptation			
	The Opt‐TBD care package and its intervention strategies will be modified for optimal fit and integration into work processes.			
	Milestone—Final refined set of implementation strategies and Opt‐TBD care package			

a CFIR guided the systematic operational steps needed to deliver the intervention practically, whereas Proctor's framework was used to assess implementation potential comprehensively. Outcomes are mostly overarching across all strategies and are defined according to Proctor's implementation outcomes.

**Figure 1:**
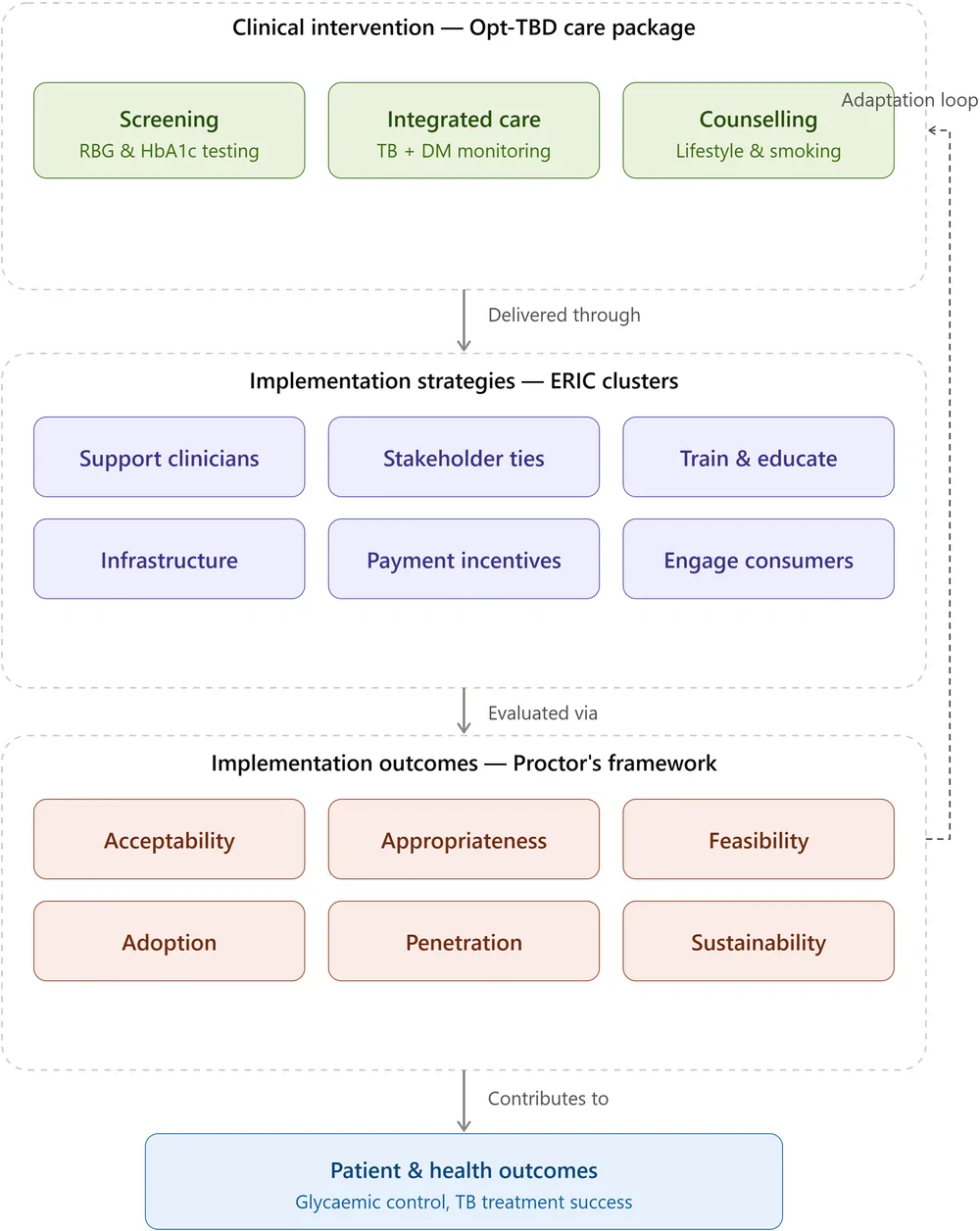
Conceptual relationship between the Opt‐TBD care package, implementation strategies, and implementation outcomes.

### 2.4 Training for TB Staff

A structured capacity‐building programme was designed to train TB staff on integrating diabetes screening and management within TB services, in line with the implementation strategies outlined in Table 1. Training sessions were based on the Opt‐TBD training guides and manuals and covered: familiarisation with the Opt‐TBD care package, diagnostic tests and criteria for diabetes, using a glucometer to monitor and record blood glucose levels, drawing, storing and transporting blood for HbA1c, counselling the patients and monitoring and managing the patients during follow‐up visits. The training was conducted over a 2‐week period, after which the integrated care package was implemented across the four TB facilities.

### 2.5 Study Outcomes

Implementation potential was explored through six implementation outcomes derived from Proctor's framework, as summarised in Table 2.

**Table 2:** Operationalisation of Proctor's implementation outcomes used to explore the implementation potential of Opt‐TBD.

Implementation Outcome	Context‐specific description
Acceptability	Perceptions of TB providers and patients regarding the suitability, usability and satisfaction with the Opt‐TBD package.
Appropriateness	The perceived fit, relevance, and compatibility of Opt‐TBD with existing workflows in BMUs and healthcare settings.
Feasibility	The extent to which the Opt‐TBD package could be successfully implemented within routine TB care at BMUs.
Adoption	The initial uptake and willingness of TB staff and facilities to incorporate Opt‐TBD into routine practice.
Penetration	The degree to which Opt‐TBD components became integrated into routine service delivery within participating BMUs.
Sustainability	The likelihood that Opt‐TBD will continue to be implemented over time within the health system.

### 2.6 Sampling and Recruitment for Qualitative Interviews

The Opt‐TBD care package was intended for adults aged ≥ 18 years with newly diagnosed active pulmonary drug‐sensitive TB within the previous 4 weeks and confirmed comorbid diabetes. Patients with severe or unstable medical conditions, pregnancy or active breastfeeding, or severe mental health conditions were excluded from receiving the care package. Consequently, patient interview participants were purposively drawn from those who met these eligibility criteria, received the Opt‐TBD care package, and provided informed consent. Healthcare providers were eligible for an interview if they were directly involved in delivering the intervention.

We included both healthcare providers involved in implementing Opt‐TBD (medical officers [MOs], DOTS Strategy Facilitators and district focal persons) and TB patients diagnosed with comorbid diabetes. After receiving the Opt‐TBD care package, the patient was provided with a detailed information sheet regarding the interview process along with a consent form. Patients were given sufficient time to understand the information and ask questions. Research Assistants provided verbal explanations of the process, ensuring the patients' comprehension of in‐depth interviews. A copy of the signed consent form was provided to the patient. Interviews were conducted by the research assistant accompanied by a note taker at the respective BMUs in a dedicated research space, ensuring patients' comfort and privacy. We conducted 12 interviews with patients and 6 interviews with healthcare providers, purposively selected to ensure diverse representation by gender, age and healthcare setting. For healthcare providers, gender distribution reflected the staffing composition of the participating BMUs. This sample size was determined based on principles of information power and anticipated data saturation, enabling us to capture rich, contextually varied insights into the implementation potential across primary, secondary and tertiary TB facilities in two provinces of Pakistan (Table 3). Sample adequacy was considered using Malterud et al.'s concept of information power. The study had a focused aim; participants were specifically selected because they had directly received or delivered Opt‐TBD; the enquiry was informed by established implementation frameworks; the interviews generated rich, context‐specific accounts, including repeated interviews with patients; and Framework Analysis enabled systematic comparison across participants, facilities and, for patient data, interview time points. Taken together, these characteristics provided sufficient information power to address the study objectives [25].

**Table 3:** Sampling plan for in‐depth interviews with service providers and service users.

Province	Hospitals	Patients with TB–diabetes multimorbidity (18 years and above)		TB staff (DOTS Strategy Facilitator, medical officer and district focal person)	
		Men	Women	Men	Women
Khyber Pakhtunkhwa	BMU 1 (Tertiary)	1	1	1	0
	BMU 2 (Primary)	2	2	2	0
	BMU 3 (Secondary)	0	2	1	1
Punjab	BMU 4 (Primary)	2	2	0	1

### 2.7 Data Collection

Trained research assistants conducted semi‐structured patient interviews at two time points, Months 3 and 6 after initiation of Opt‐TBD delivery, aligning with routine patient follow‐up visits. Healthcare providers were interviewed towards the end of the implementation period. Interviews were guided by topic guides specifically developed using Proctor's implementation framework and informed by the CFIR's Process domain. These topic guides explicitly explored multiple dimensions of implementation, including acceptability, appropriateness, feasibility, adoption, penetration and sustainability. CFIR constructs specifically informed practical questions around the operational implementation processes (e.g., stakeholder engagement, adaptability and resource constraints), enriching the depth of qualitative data collection.

A common framework‐based topic guide was used across both rounds to preserve conceptual comparability. The Month 6 guide retained the core questions used at Month 3 and added time‐specific probes on continuity, changes in workflow, resolution of earlier barriers, routinisation, penetration and sustainability.

At Month 3, interviews primarily focused on participants’ initial experiences of implementing the Opt‐TBD care package through the contextually designed strategies. Discussions explored perceptions of early barriers, facilitators, appropriateness, acceptability and feasibility, explicitly capturing recommendations for mid‐course refinements. By Month 6, interviews explored perceptions around sustainability, penetration of the intervention into routine practices, and any changes to workflows among healthcare staff following training and support. Interviews further examined whether initial barriers had been resolved and how effectively the intervention had become integrated within routine TB care processes.

Interviews were conducted by trained research assistants with health‐research experience, accompanied by a trained note‐taker. Interviews were conducted in each participant's preferred local language, including Urdu or Pashto, as appropriate. Topic guides were translated into the relevant local languages, and interviewers used culturally familiar wording and probes to support participant understanding. Interviews were conducted inside a private room within the BMU, ensuring confidentiality and comfort. Written informed consent was obtained prior to all interviews.

### 2.8 Data Analysis

Audio recordings were transcribed verbatim and translated into English to ensure accuracy and consistency. A Framework Analysis approach (familiarisation, identifying a thematic framework, indexing, charting and mapping/interpretation), guided by Proctor's implementation outcomes framework, was used a priori along with sub‐themes derived inductively from the data [25]. This analytical approach allowed for structured examination of multiple key implementation outcomes, including acceptability, appropriateness, feasibility, adoption, penetration, and sustainability.

Following data familiarisation, the research team reviewed transcripts and field notes to identify emerging patterns. Two members of the research team independently coded the transcripts, compared and discussed them to reach consensus. As data collection continued across two time points, new codes were added and refined iteratively as transcripts were analysed. The coding matrix was managed manually in Microsoft Excel, consistent with established approaches to Framework Analysis [25].

Once coding was finalised, transcripts were systematically indexed according to individual interviews, and data were charted into a matrix format to facilitate comparisons across participant groups, interview time points and study settings. The matrices were reviewed, and thematic patterns were identified, and differences and similarities across participant groups were identified. Analytic memos were maintained throughout the analysis to document coding decisions, refinements and researcher reflections, ensuring transparency and rigour. Findings were organised explicitly according to Proctor's outcomes framework, detailing stakeholder perceptions, implementation challenges, and areas of early adoption and embedding within routine practice. Finally, synthesised data were used to identify key facilitators and barriers to the integration of the Opt‐TBD care package.

## 3 Results

### 3.1 Overview of Participants

Recruitment of participants for qualitative interviews varied across facility levels. Purposive sampling was applied flexibly within each facility to capture a range of available and eligible participants rather than aiming for equal recruitment targets across each facility level.

A total of 18 participants (12 patients and 6 healthcare providers) from the four TB facilities participated in qualitative interviews. Sociodemographic characteristics are summarised in Table 4 and Table 5.

**Table 4:** Sociodemographic characteristics of patients with TB and diabetes multimorbidity.

Characteristics	*N* (%)
Sex	
Male	5 (41.66)
Female	7 (58.33)
Age (years; mean, range)	43.5 (24–55)
Education	
No education	10 (83.3)
Up to 10 years	2 (16.7)
Other	0
Employment	
Unemployed	8 (66.7)
Self‐employed	2 (16.7)
Employed	2 (16.7)
Monthly household income	
< 20k	5 (41.7)
20k–40k	6 (50.0)
> 40k	1 (8.3)
Marital status	
Single	0
Married	12 (100.0)
Location	
Urban	9 (75.0)
Rural	3 (25.0)

*Note:* Age is presented as mean, range, and the remaining variables are presented as *n* (%)

**Table 5:** Sociodemographic characteristics of healthcare providers.

Characteristics	*N* (%)
Roles	
District Focal Person	1 (16.7)
Medical Officer	2 (33.3)
DOTS Strategy Facilitator	3 (50.0)
Sex	
Male	4 (66.7)
Female	2 (33.3)
Age (years; mean, range)	37.3 (32–42)
Length of time as a Healthcare provider (years; mean, range)	14.83 (10–19)
Length of time in current role (years; mean, range)	9.75 (2–18)

*Note:* Age, length of time as a healthcare provider and length of time in current role are presented as Mean, range, and the remaining variables are presented as *n* (%).

*Patients* (*n* = 12): Included 5 males and 7 females with TB–diabetes multimorbidity. Participants had a mean age of 43.5 (24–55) years; most were uneducated (*n* = 10), unemployed (*n* = 8), all were married, and predominantly urban residents (*n* = 9). Eleven of the 12 participants (91.7%) reported a monthly income below PKR 40,000, including 5 (41.7%) below PKR 20,000. For context, the statutory minimum wage in both Khyber Pakhtunkhwa and Punjab was PKR 32,000 per month during the study period, indicating the relatively low socioeconomic position of much of the study sample [26].

*Healthcare providers* (*n* = 6): Included DOTS Strategy Facilitators (*n* = 3), MOs (*n* = 2) and a district focal person (*n* = 1). Participants had a mean age of 37.3 (32–42) years, averaging 14.8 years of professional experience and 9.7 years in their current roles.

### 3.2 Main Themes

The analysis examined participants' experiences of the contextually tailored Opt‐TBD package and its associated implementation strategies to integrate diabetes care within routine TB services. Participant accounts were systematically charted and synthesised within Proctor's implementation outcome framework to identify factors influencing acceptability, appropriateness, feasibility, adoption, penetration and sustainability of the Opt‐TBD package. Data‐driven subthemes captured facilitators, barriers, and contextual factors affecting implementation. Illustrative quotations from participants are presented throughout the findings, with a condensed summary of key themes and representative quotations provided in Table 6.

**Table 6:** Key themes, sub‐themes and illustrative quotations.

Theme	Sub‐theme	Quote ID	Representative quote/s (participant)
Acceptability	Perceived value of integrated services	P1 FP1 DOTS3 FP1 MO1	‘I know the test is charged for a big amount of 1400 rs or 1500 rs, he (DOTS Strategy Facilitator) did my emergency test (RBG) and then the big test (HbA1c), I received this facility within one place, I have not seen this anywhere, that you go to a place for treatment of one issue and you receive treatment for two of your issues. This was the first time; I got very satisfied from here (TB facility)’. (Patient P1, 34 years, Male) ‘I think that through this programme (care package), they (patients) have better awareness about diabetes and TB’. (District TB‐Focal Person FP1, Peshawar) ‘When such (poor) patients are screened and diagnosed free of cost, and medicine provided to them for both TB and diabetes, such patients continue with adherence, and get treated completely’. (DOTS Strategy Facilitator DOTS3, Secondary level‐BMU) ‘It (care package) is beneficial, because I said that it is not only for TB, TB is finished after 6 months but with the use of medicine and with the lifestyle modification, he can live a better life. If the patient is sensible and educated, he can spread awareness about diabetes in his family and community’. (District TB‐Focal Person FP1, Peshawar) ‘It is a very good programme, and due to this, we were able to detect some patients in the earlier stage and follow up on already diagnosed diabetic patients. HbA1c can be done in this programme. It's overall very beneficial’. (Medical Officer MO1, Secondary level‐BMU)
	Patient comfort with testing	P2 P3 DOTS2 P2	‘The doctors are supportive and cooperative… the administration of sugar tests was very convenient, I became happy when my tests were done’. (Patient P2, 55 years, Female) They (DOTS Strategy Facilitator) ask me that we would also test your blood. So I thinks; what? why are they doing this? Why further? (Patient P3, 55 years, Female) ‘The patient was diabetic but for many months he had not done RBG because of poverty… his RBG and HbA1c was done. So that was a good thing’. (DOTS Strategy Facilitator DOTS2, Primary level‐BMU) When I come here for my treatment, there are some costs involved, especially for travel. But I'm grateful that you cover the travel costs, as that makes a big difference for me. (Patient P2, 55 years, Female)
	Overall acceptability (Months 3–6)	P1	‘When I started taking those medicines, my sugar dropped very low. I used to feel dizzy and nauseated. For about 10 to 15 days I had pain in my legs. I left using the medicine and did not use it again because I was having a lot of problems’. (Patient P1, 34 years, Male)
Appropriateness	Fit with existing TB workflow	DOTS2 FP1 DOTS1	‘It (care package) is easy to give in our facility. The doctor comes from below and reviews the patient. We (DOTS Strategy Facilitator) do the counselling. When we (DOTS Strategy Facilitator) do HbA1c, then our District TB Assistant (DTA)^a^ takes the sample. The patient does not have any problem’. (DOTS Strategy Facilitator DOTS2, Primary level‐BMU) ‘TB patients, when diagnosed on the first visit, then on the second month, on the fifth and sixth. The patient does not have any problems. The visits and follow‐ups are done in the same way. So, the patient is adhering to the components. Yes, the components are coherent and it is adhering to each other’. (District Focal Person FP1, Peshawar) ‘There are some obstacles, let me explain, as you know RBS is a very rapid test done on the spot, but as far as HBA1c is concerned it takes time and patients have to wait outside. As a result, many patients gather here such as ward patients, attendants, and TB patients, which creates a burden on us the health professionals working around me as the DOTS Strategy Facilitator room is inside the medical ward’. (DOTS Strategy Facilitator DOTS1, Tertiary level‐BMU)
	Cultural relevance	P2 P3 P1	‘I practiced abstinence (restricted diet), I avoided spicy food, and ate food that included yakhni (soup) and vegetables’. (Patient P2, 55 years, Female) ‘(Name of DOTS) briefly explained to me that they also offer diabetes treatment along with TB treatment. He tested my RBS, which was 400 and conducted a blood test (HbA1c) that came back positive. He counselled me on my diet, daily routine, and physical activities, and advised me to avoid sugar. (Name of DOTS) provided me with a prescription and guidance about the free treatment available at (Tertiary hospital). I went to (Tertiary hospital) and received my medication free of cost’. (Patient P3, 55 years, Female) ‘I was shown the ways to control my sugar, (such as) not consuming sweets or practicing abstinence, showed me the right way; what to eat, and what to avoid. All the staff is good here but no matter how much I thank the name of the person (DOTS Strategy Facilitator) it will be less’. (Patient P1, 34 years, Male)
	Overall appropriateness (Months 3–6)	P6	‘TB is getting better, but no relief from sugar. I am experiencing excruciating agony, unable to walk normally for an entire day. They should send me to a reputable diabetic physician; he should prescribe medication, administer insulin injections, or do whatever else is necessary to restore my mobility and overall health’. (Patient P6, 32 years, Female)
Feasibility	Resource Availability	FP1 MO1 FP1 DOTS2 P2 DOTS2 DOTS2 MO2 DOTS1	‘There were no issues related to resources. No challenges in the delivery of care package concerning finance, resources, or communication’. (District TB‐Focal Person FP1, Peshawar) ‘The biggest issue was the cost of HbA1c, which you guys have catered to, so it is a big strength that you provided equipment support in the form of Glucometers. Without equipment, we would not be able to implement the care package. Patients had affordability issues, not every patient was able to get HbA1c done. This was the strength that everything was provided to us, glucometer is available on spot, so RBS is done there and then and we faced no issue in this’. (Medical Officer MO1, Secondary level‐BMU) ‘We suggest that there should be 2 to 3 more tests, especially lipid profile, for obese patients, or especially if the female is having urine RE. So, if lipid profile and urine RE are there, then it further helps us to improve the outcomes of TB and diabetes patients’. (District TB‐Focal Person FP1, Peshawar) ‘No, we did not feel any lack of resources that may have delayed the package from one day to the other. There was no issue faced with regard to the kits, the strips, or anything else’. (DOTS Strategy Facilitator DOTS2, Primary level‐BMU) ‘The most helpful part of this treatment is that all diagnostic tests and medications are provided for free. This approach is commendable because it involves conducting our tests, providing us with free medication for both tuberculosis and diabetes, and offering incentives to ensure we visit regularly. I am confident that I will make a full recovery soon’. (Patient P2, 55 years, Female) ‘Patients also come due to incentives particularly daily wagers. Patients commit but do not show up for follow‐up due to personal reasons such as having their own work, travel allowance motivates them to visit’. (DOTS Strategy Facilitator DOTS2, Primary level‐BMU) ‘The patient will come only for the incentives and also telling other patients leading to a problem and at each visit they will be asking for incentives believing that the health care provider kept it in their pocket. Free tests are sufficed leading to patient satisfaction’. (DOTS Strategy Facilitator DOTS2, Primary level‐BMU) ‘Give incentives to the family members. I think that especially if incentives are given, then the family members will agree that there is no financial burden on them, then they will get them (patients) treated, especially the old age patients, they have more problems therefore incentives should be given’. (Medical Officer MO2, Primary‐Level BMU) ‘Totally you have to talk with our administration that we need a small room or cabin‐type room where we take blood from the patient, everyone sees what is going on and why the blood is being taken. So, then there is a different stigma that there might be an issue, why the blood is being taken from him. From a lady, then he has to take it from the side behind a curtain and take blood from the muscle or from somewhere else, then it is better if there is a separate place, if a female comes in between, then where will they take her? So, if you get a small cabin‐type or a room‐type place, then it is better. If you have to do counselling then privacy is a must for that, but I do not have those things available’. (DOTS Strategy Facilitator DOTS1, Tertiary level‐BMU)
	Referral mechanism	DOTS2 DOTS2 P1 FP1	‘They (lab) send the reports (HbA1c test) to us online’. (DOTS Strategy Facilitator DOTS2, Primary level‐BMU) ‘But the referral issue, you remember, (name of patient), we sent him to the hospital, but instead of sugar OPD, he went to other OPD. So, this is an issue of referral. So, if you can do something like this that instead of the OPD, they should be referred and checked in the ward. So, the patient will not have any difficulty’. (DOTS Strategy Facilitator DOTS2, Primary level‐BMU) ‘I asked about the sugar doctor and they told me to go to that room. There was a label but I was illiterate,… I went there on the instructions given here but some doctors do not help poor people, they only fill the prescription and say buy this medicine and go. We are helpless people and cannot argue with anyone’. (Patient P1, 34 years, Male) ‘If they need further care, i.e if their HbA1c level is so high that they cannot manage, then we also send them to special care or medical specialist. It would be better if there is a young person, such as young associate or assistant professor level person, he can focus more on these things (Tb‐diabetes comorbidity management) and communicate better’. (District TB‐Focal Person FP1, Peshawar)
	Training and staff capacity	DOTS2 FP1 MO2	‘It will be good if refresher trainings are given because sometimes there is an issue with the patient and we get confused so we go downstairs to the doctor so if training is given it will be good so that if we do not understand anything or there is something new it will get clear to us’. (DOTS Strategy Facilitator DOTS2, Primary level‐BMU) ‘So, further training should be done from time to time because a person should be upgraded along with his health. I think this is enough for now’. (District TB Focal Person FP1, Peshawar) ‘You get us trained a little. It will be good for us too. So, it happens that everyone comes to the same page. When a staff member and a medical officer will give the same thing, then everyone will automatically come to the same page’. (Medical Officer MO2, Primary level‐BMU).
Adoption	Early uptake	P2 DOTS1 FP1 FP1	‘I practiced abstinence (restricted diet), I avoided spicy food, and ate food that included yakhni (soup) and vegetables. I took my prescribed medication on time and followed a restricted diet, I did exercise’. (Patient P2, 55 years, Female) ‘Burden on staff increase in terms of time and additional task of RBG and HbA1c and patients wait but its manageable on daily basis. The burden is placed, if there is a new patient, the registration will take at least 5 to 10 min. I must do RBG screening as per my role in the project which also takes time. So, there is an extra burden due to extra duties associated with the integrated care, but I will say it's not a problem for me. Additionally, I take blood samples of patients for HbA1c and send it to the nearby laboratory for a test. So, it's manageable and I manage it on a daily basis.’ (DOTS Strategy Facilitator DOTS1, Tertiary level‐BMU). ‘In the beginning, there was a problem but now it is a regular routine. In the beginning, there was little problem in diagnosing (diabetes), then doing consultation and then to send him to the lab of tertiary care and reports, and then to communicate further. But now it is channelised, in the BMUs, it will be easily used in the routine’. (District TB Focal Person FP1, Peshawar) ‘We have a lot of patients on Monday, Tuesday, and Wednesday. We have a problem in these days. Patients have high flow and OPD is higher. So, it takes a little extra effort because we have an OPD to run… listening to them and counseling them takes extra time’. (District TB Focal Person FP1, Peshawar)
	Leadership and programme support	MO2 *MO2*	‘Upon receiving a mission or direction, consistently follow and continue to implement the guidance from NTP as needed’. (Medical Officer MO2, Primary level‐BMU) ‘It is obvious that when we get a mission or a direction, then we will bond with it. Then it will be in our guidelines at every place. And then we follow it. There are still a lot of things that NTP keeps telling us from time to time, so we try to implement it’. (Medical Officer MO2, Primary level‐BMU)
Penetration	Integration into daily routines	FP1 MO1 DOTS2	‘As a general health worker, I can help the patients with diabetes and treat both diseases and patients and if the same patient can get help in their family and society then why not, I think it is a matter of honour for us and I would like that this should be started in other BMUs and further training and sessions should be done. I think this is a very good thing and we should do it’. (District TB‐Focal Person FP1, Peshawar) ‘The programme is extremely important due to increasing TB prevalence and diabetes among TB patients. It is ready for full implementation, making TB and diabetes management easier and benefiting patients, as previously suggested’. (Medical Officer MO1, Secondary level‐BMU) ‘In our routine care this change has taken place, who are not recruited in your project the extra pulmonary so we do their counselling as well and ask them about their diabetes, we ask whether any one at home have diabetes, or do you have such symptoms or any of your parents have diabetes, or do you get thirsty a lot or frequently experience urination at night, so this is one thing for us, that we have included this in the TB counselling. It is easy; there is no problem for us. There were some issues in the beginning, because we would forget certain things but now gratefully counselling and everything is done’. (DOTS Strategy Facilitator DOTS2, Primary level‐BMU)
	Collaboration with specialised care	DOTS1 DOTS1	‘In case of uncontrolled sugar, they are referred to an endocrinologist, a medical specialist, or a tertiary care hospital. They guide them further. During follow ups, feedback is taken from the patients about their sugar and medicines. A very good personal experience with the program’. (DOTS Strategy Facilitator DOTS1, Tertiary level‐BMU) ‘We have a little problem in the laboratory because it takes 3‐4 h in processing. So, if our liaison is formed with the laboratory, they do the HbA1c when they receive it, they do it early so the patient would not have to wait for long and don't come again I used to do it like this that I used to write the number of the patient and the share the report on the WhatsApp. So, then for the physicians they used to take printouts. I did not call the patient again. I used to send the report on WhatsApp’. (DOTS Strategy Facilitator DOTS1, Tertiary level‐BMU)
	Overall penetration (Months 3–6)	P8 P6	‘The medications (sugar) are expensive, which can be a burden, especially during times of economic inflation. While the program helps those with financial hardships, patients still have to pay for medications, adding to their financial strain’. (Patient P8, 52 years, Male) ‘Overall, everything was good quality‐wise, I had no problem except that the waiting part was a bit issue, gratefully Alhamdulillah everything was perfect’. (Patient P6, 32 years, Female)
Sustainability	Financial incentives and continuity	DOTS2 FP1 FP1 P4 DOTS1	‘The incentives you are giving us, i.e. also possible. But we do the work anyway. So, one positive thing is that a person is encouraged with incentives. Like with the District TB Assistant, there were some problems but now it has been resolved. So, it makes the delivery process easier’. (DOTS Strategy Facilitator DOTS2, Primary level‐BMU) ‘So, personally, our (name of doctor) and DTO are committed. If the project is done or not, we will keep consulting the patient about TB and diabetes. If the patient is not cured, we will keep on guiding him. If the patient is sick and diagnosed with TB, we will give him some lifestyle modification and counselling’. (District TB Focal Person FP1, Peshawar) ‘I think financial assistance of the healthcare staff is not a hindrance’. (District TB Focal Person FP1, Peshawar) ‘I also remember that each time I visited, you provided money to cover the cost of traveling to the TB centre, which was a huge help. This level of support, both financially and medically, makes it much easier for patients like me to adhere to the treatment and get better. All these components—the free testing, the follow‐ups, and the travel assistance—have made a big difference in my recovery and are, in my view, the strongest parts of the care package’. (Patient P4, 45 years, Male) ‘We will have to see what is going on in the procurement or what does the government do because all the medicines of our TB are coming from outside, it is coming from (name of fund). We do not have any kind of resources from the national government or the provincial government. Everything is done from outside (abroad). So, this is the situation. If the government takes it up with the professional people's support, if they get some benefit, if they get some package, then that would be a benefit for them. It will be a source of attraction. Otherwise, it is very difficult for our government to take it up’. (DOTS Strategy Facilitator DOTS1, Tertiary level‐BMU)
	Long‐term embedding in TB program	DOTS3 DOTS1 MO2 DOTS2	‘This is an extremely important programme that needs to be implemented for a longer period of time. This is due to the fact that TB is becoming more prevalent every day, and diabetes is also extremely prevalent among those who have TB. It is ready for full implementation and nothing is missing. I have provided some suggestions in the past’. (DOTS Strategy Facilitator DOTS3, Secondary level‐BMU) ‘If it (care package) is merged into the TB Control Program, then it will be beneficial’. (DOTS Strategy Facilitator DOTS1, Tertiary level‐BMU) ‘The NTP (National TB Control Programme) people take it (care package) seriously. And I hope that God willing, they will take it seriously in the future’. (Medical Officer MO2, Primary level‐BMU) ‘It's a good thing If you implement this with us, like it is done in our facility, and in other hospitals, and diagnostic centres, it will be a good thing; this will facilitate the patient even more. He will have awareness; he will change his lifestyle. He will take care of other people as well. Like, he will stop smoking and engage in other activities. He will change his lifestyle and reduce his diabetes’. (DOTS Strategy Facilitator DOTS2, Primary level‐BMU)

a DTA stands for district TB assistant, a field staff member who works at the local level to collect sputum from their assigned BMUs and transport it to the health facility equipped with a GeneXpert machine for testing.

To explore the implementation potential of Opt‐TBD, findings were organised using six outcomes from Proctor's implementation outcomes framework: acceptability, appropriateness, feasibility, adoption, penetration and sustainability. Within these higher‐order domains, subthemes were developed inductively from participants' accounts.

A condensed version of key themes with representative quotes is presented in Table 6.

### 3.3 Acceptability

This theme explored TB providers and patients' views on the suitability, usability and satisfaction with the Opt‐TBD package.

#### 3.3.1 Sub‐Theme 1A: Perceived Value of Integrated TB–Diabetes Services

Patients appreciated the integrated TB–diabetes care within the same facility, noting improved adherence to TB treatment, earlier diabetes detection, and overall health benefits. Provision of free diagnostic tests (RBG, HbA1c) reduced financial barriers, while integrated services minimised logistical challenges, treatment delays, and patient stress (Quotes P1).

I know the test is charged for a big amount of 1400 rs or 1500 rs, he (DOTS Strategy facilitator) did my emergency test (RBG) and then the big test (HbA1c), I received this facility within one place, I have not seen this anywhere, that you go to a place for treatment of one issue and you receive treatment for two of your issues. This was the first time; I got very satisfied from here (TB facility).

(Patient P1, 34 years, Male)

District TB Focal Persons particularly highlighted the value of timely diabetes diagnosis through routine diabetes screening within TB services. More broadly, healthcare providers emphasised that standardised free‐of‐cost diabetes screening within routine TB care facilitated timely diabetes diagnosis, while targeted counselling enhanced patient awareness, lifestyle modification and treatment adherence, potentially reducing complications, hospital readmissions and overall health system burden. Embedded referral mechanisms supported continuity of diabetes care post‐TB treatment (Quotes FP1, DOTS3).

I think that through this programme (care package), they (patients) have better awareness about diabetes and TB.

(District TB‐Focal Person FP1, Peshawar)

When such (poor) patients are screened and diagnosed free of cost, and medicine provided to them for both TB and diabetes, such patients continue with adherence, and get treated completely.

(DOTS Strategy Facilitator DOTS3, Secondary level‐BMU)

It (care package) is beneficial, because I said that it is not only for TB, TB is finished after 6 months but with the use of medicine and with the lifestyle modification, he can live a better life. If the patient is sensible and educated, he can spread awareness about diabetes in his family and community.

(District TB‐Focal Person FP1, Peshawar)

Providers also viewed the integrated approach as beneficial, particularly for early diabetes detection and follow‐up, and supported its wider implementation within the national TB programme (Quote MO1).

It is a very good programme, and due to this, we were able to detect some patients in the earlier stage and follow up on already diagnosed diabetic patients. HbA1c can be done in this programme. It's overall very beneficial.

(Medical Officer MO1, Secondary level‐BMU)

#### 3.3.2 Sub‐Theme 1B: Patient Comfort With Additional Testing

Patients generally viewed the additional diabetes testing positively, particularly appreciating the rapid and convenient RBG test. However, the HbA1c test posed challenges due to longer processing times, causing extended waits and lost wages. Patients also reported uncertainty regarding the purpose of the HbA1c test, highlighting a need for improved patient education (Quotes P2, P3)

The doctors are supportive and cooperative… the administration of sugar tests was very convenient, I became happy when my tests were done.

(Patient P2, 55 years, Female)

They (DOTS Strategy Facilitator) asked me that we would also test your blood. So I thinks; what? why are they doing this? Why further?

(Patient P3, 55 years, Female)

Nevertheless, integrating free RBG and HbA1c testing within TB services notably reduced logistical barriers. Blood samples were transported by district TB assistants (DTA) or designated staff, with online sharing of results, minimising patient travel and improving follow‐up adherence. Additionally, travel allowances provided to patients helped alleviate financial constraints related to repeated facility visits (Quote DOTS1, P2).

The patient was diabetic but for many months he had not done RBG because of poverty… his RBG and HbA1c was done. So that was a good thing.

(DOTS Strategy Facilitator DOTS2, Primary level‐BMU)

When I come here for my treatment, there are some costs involved, especially for travel. But I'm grateful that you cover the travel costs, as that makes a big difference for me.

(Patient P2, 55 years, Female)

Acceptability of the Opt‐TBD package remained consistently high across both timepoints. At Month 3, patients primarily appreciated receiving integrated TB and diabetes services within the same facility, particularly the availability of free diagnostic tests, medicines, counselling and supportive healthcare providers. By Month 6, these positive perceptions were sustained, with patients describing improved health, greater confidence in managing diabetes, and continued satisfaction with the integrated care approach. Despite these positive experiences, some patients continued to report concerns related to diabetes medication side effects, treatment costs, persistent symptoms, and limited improvement in glycaemic control, indicating that high acceptability did not eliminate all concerns (Quote P1).

When I started taking those medicines, my sugar dropped very low. I used to feel dizzy and nauseated. For about 10 to 15 days I had pain in my legs. I left using the medicine and did not use it again because I was having a lot of problems.

(Patient P1, 34 years, Male)

### 3.4 Appropriateness

This theme examines how smoothly the care package integrates within the TB routine, any logistical challenges encountered and the cultural relevance of the care package.

#### 3.4.1 Sub‐Theme 2A: Fit With Existing TB Workflow

Healthcare providers reported that the Opt‐TBD care package integrated smoothly into routine TB workflows with minimal disruption. Diabetes screening and counselling were easily incorporated into routine monthly TB follow‐up visits, requiring no major changes to existing practices. Clearly defined staff roles—such as DOTS Strategy Facilitators conducting RBG and HbA1c tests, recording results, and providing counselling, and MOs managing referrals for uncontrolled diabetes while DTAs transported the samples to lab—enabled efficient implementation and effective coordination among team members (Quotes DOTS2, FP1).

It (care package) is easy to give in our facility. The doctor comes from below and reviews the patient. We (DOTS Strategy Facilitator) do the counselling. When we (DOTS Strategy Facilitator) do HbA1c, then our District TB Assistant (DTA)* takes the sample. The patient does not have any problem.

(DOTS Strategy Facilitator DOTS2, Primary level‐BMU)

TB patients, when diagnosed on the first visit, then on the second month, on the fifth and sixth. The patient does not have any problems. The visits and follow‐ups are done in the same way. So, the patient is adhering to the components. Yes, the components are coherent and they adhere to each other.

(District Focal Person FP1, Peshawar)

However, providers identified logistical challenges, notably the longer processing times required for HbA1c testing, which led to patient crowding in facilities with limited space and high patient volume, placing additional burdens on staff managing concurrent TB and diabetes services (Quote DOTS1).

There are some obstacles, let me explain, as you know RBS is a very rapid test done on the spot, but as far as HBA1c is concerned it takes time and patients have to wait outside. As a result, many patients gather here such as ward patients, attendants, and TB patients, which creates a burden on us the health professionals working around me as the DOTS Strategy Facilitator room is inside the medical ward.

(DOTS Strategy Facilitator DOTS1, Tertiary level‐BMU)

#### 3.4.2 Sub‐Theme 2B: Cultural Relevance

Patients found the integrated lifestyle counselling culturally appropriate, as it reflected local dietary norms and daily routines. Counselling on diet, physical activity and smoking cessation was tailored to local contexts, promoting practical and sustainable lifestyle modifications without imposing unrealistic dietary restrictions. Rather than requiring patients to completely eliminate certain foods from their diet, healthcare providers encouraged them to consume healthier options, such as vegetables and soups, while reducing or avoiding foods that aggravated their condition, including spicy and oily foods and bakery items. This personalised approach empowered patients to make informed and manageable lifestyle changes for improved diabetes control.

Additionally, counselling incorporated holistic health messages, emphasising physical activity and stress management. Diagnostic tests were performed respectfully, with clear communication regarding results, treatment options, and the availability of medications. This culturally sensitive and considerate approach enhanced patient trust, comfort and active engagement with integrated TB–diabetes care (Quotes P2, P3, P1).

I practiced abstinence (restricted diet), I avoided spicy food, and ate food that included yakhni (soup) and vegetables.

(Patient P2, 55 years, Female)

(Name of DOTS) briefly explained to me that they also offer diabetes treatment along with TB treatment. He tested my RBS, which was 400 and conducted a blood test (HbA1c) that came back positive. He counselled me on my diet, daily routine, and physical activities, and advised me to avoid sugar. (Name of DOTS) provided me with a prescription and guidance about the free treatment available at (Tertiary hospital). I went to (Tertiary hospital) and received my medication free of cost.

(Patient P3, 55 years, Female)

I was shown the ways to control my sugar, (such as) not consuming sweets or practicing abstinence, showed me the right way; what to eat, and what to avoid. All the staff is good here but no matter how much I thank the name of the person (DOTS Strategy Facilitator) it will be less.

(Patient P1, 34 years, Male)

The perceived appropriateness of the Opt‐TBD package remained stable over time. Earlier patients valued the convenience of receiving TB treatment, diabetes screening, monitoring and counselling through a single care pathway. However, some participants felt that additional diabetes specialist input was required when glycaemic control remained suboptimal, suggesting opportunities to further strengthen the appropriateness of the care model (Q P6). Later, patients demonstrated a stronger understanding of the importance of managing TB and diabetes concurrently and continued to perceive the integrated approach as relevant to their healthcare needs.

TB is getting better, but no relief from sugar. I am experiencing excruciating agony, unable to walk normally for an entire day. They should send me to a reputable diabetic physician; he should prescribe medication, administer insulin injections, or do whatever else is necessary to restore my mobility and overall health.

(Patient P6, 32 years, Female)

### 3.5 3. Feasibility

The theme investigated resource availability, the effectiveness and the limitations of the referral mechanism and the importance of capacity building of healthcare providers in implementing the Opt‐TBD care package.

#### 3.5.1 Sub‐Theme 3A: Resource Availability

Healthcare providers reported that the Opt‐TBD care package was adequately resourced, with sufficient supplies of glucometers and testing kits, enabling smooth service delivery. Providers recommended further optimisation through additional resources, including lipid profile tests for obese patients, routine urine examinations for women, blood pressure monitors and diet charts (Quotes FP1, MO1).

There were no issues related to resources. No challenges in the delivery of care package concerning finance, resources, or communication.

(District TB‐Focal Person FP1, Peshawar)

The biggest issue was the cost of HbA1c, which you guys have catered to, so it is a big strength that you provided equipment support in the form of Glucometers. Without equipment, we would not be able to implement the care package. Patients had affordability issues, not every patient was able to get HbA1c done. This was the strength that everything was provided to us, glucometer is available on spot, so RBS is done there and then and we faced no issue in this.

(Medical Officer MO1, Secondary level‐BMU)

We suggest that there should be 2 to 3 more tests, especially lipid profile, for obese patients, or especially if the female is having urine RE. So, if lipid profile and urine RE are there, then it further helps us to improve the outcomes of TB and diabetes patients.

(District TB‐Focal Person FP1, Peshawar)

Staff noted consistent availability of testing kits, strips and medical equipment, highlighting this reliability as essential for uninterrupted implementation (Quote DOTS2). Travel allowances supported patient retention and improved follow‐up, particularly among those with limited income. Healthcare providers highlighted affordability as an important determinant of patients' continued engagement with diabetes care, with MOs placing particular emphasis on this issue. Some healthcare providers suggested extending financial support to caregivers of elderly patients to further enhance treatment adherence. Nevertheless, the consensus was that free diagnostic tests and medications sufficiently supported patient engagement and satisfaction (Quotes P2, DOTS2, MO2).

No, we did not feel any lack of resources that may have delayed the package from 1 day to the other. There was no issue faced with regard to the kits, the strips, or anything else.

(DOTS Strategy Facilitator DOTS2, Primary level‐BMU)

The most helpful part of this treatment is that all diagnostic tests and medications are provided for free. This approach is commendable because it involves conducting our tests, providing us with free medication for both tuberculosis and diabetes, and offering incentives to ensure we visit regularly. I am confident that I will make a full recovery soon.

(Patient P2, 55 years, Female)

Patients also come due to incentives, particularly daily wagers. Patients commit but do not show up for follow‐up due to personal reasons such as having their own work, travel allowance motivates them to visit.

(DOTS Strategy Facilitator DOTS2, Primary level‐BMU)

The patient will come only for the incentives and also telling other patients leading to a problem and at each visit they will be asking for incentives believing that the health care provider kept it in their pocket. Free tests are sufficient leading to patient satisfaction.

(DOTS Strategy Facilitator DOTS2, Primary level‐BMU)

Give incentives to the family members. I think that especially if incentives are given, then the family members will agree that there is no financial burden on them, then they will get them (patients) treated, especially the old age patients, they have more problems therefore incentives should be given.

(Medical Officer MO2, Primary‐Level BMU)

Operational challenges were most frequently highlighted by DOTS *Strategy* Facilitators, whose day‐to‐day responsibility for conducting diabetes screening and counselling provided direct insight into infrastructure limitations. Despite adequate medical supplies, space limitations and privacy concerns were key challenges, especially in high‐volume tertiary‐level facilities. The lack of dedicated spaces for HbA1c testing and counselling sessions compromised confidentiality, led to frequent disruptions, and raised concerns about stigma related to additional blood tests. Providers emphasised the need for private spaces or enclosed areas, particularly to ensure comfort and confidentiality for female patients (Quote DOTS1).

Totally you have to talk with our administration that we need a small room or cabin‐type room where we take blood from the patient, everyone sees what is going on and why the blood is being taken. So, then there is a different stigma that there might be an issue, why the blood is being taken from him. From a lady, then he has to take it from the side behind a curtain and take blood from the muscle or from somewhere else, then it is better if there is a separate place, if a female comes in between, then where will they take her? So, if you get a small cabin‐type or a room‐type place, then it is better. If you have to do counselling then privacy is a must for that, but I do not have those things available.

(DOTS Strategy Facilitator DOTS1, Tertiary level‐BMU)

#### 3.5.2 Sub‐Theme 3B: Referral Mechanism

District TB Focal Persons highlighted the importance of clear referral pathways and coordination with specialist services to ensure continuity of diabetes care for patients requiring further management, while MOs emphasised the need for ongoing clinical follow‐up. Healthcare providers reported that a structured referral system was established, connecting TB facilities with government medical specialists or diabetologists, enabling timely specialist consultation and efficient online access to laboratory results. However, healthcare providers and patients at primary‐level facilities reported referral challenges, with patients having trouble navigating tertiary care centres and locating designated specialists. To mitigate these challenges, healthcare providers recommended direct referrals to inpatient wards and involving younger clinicians, such as associate or assistant professors, who could specifically manage TB–diabetes comorbidity (Quotes DOTS2, FP1).

They (lab) send the reports (HbA1c test) to us online.

(DOTS Strategy Facilitator DOTS2, Primary level‐BMU)

But the referral issue, you remember, (name of patient), we sent him to the hospital, but instead of sugar OPD, he went to another OPD. So, this is an issue of referral. So, if you can do something like this instead of the OPD, they should be referred and checked in the ward. So, the patient will not have any difficulty.

(DOTS Strategy Facilitator DOTS2, Primary level‐BMU)

I asked about the sugar doctor and they told me to go to that room. There was a label but I was illiterate,… I went there on the instructions given here but some doctors do not help poor people, they only fill the prescription and say buy this medicine and go. We are helpless people and cannot argue with anyone.

(Patient P1, 34 years, Male)

If they need further care, i.e if their HbA1c level is so high that they cannot manage, then we also send them to special care or medical specialist. It would be better if there is a young person, such as a young associate or assistant professor level person, who can focus more on these things (TB‐diabetes comorbidity management) and communicate better.

(District TB‐Focal Person FP1, Peshawar)

#### 3.5.3 Sub‐Theme 3C: Training and Staff Capacity

Capacity‐building through targeted training was highly valued by healthcare providers, equipping them with skills necessary to integrate diabetes care effectively into routine TB services. Providers highlighted the importance of ongoing refresher training to maintain consistent service delivery, improve problem‐solving and reinforce knowledge. Alignment between MOs and DOTS Strategy Facilitators was identified as critical to the successful and sustained implementation of the intervention within the existing TB control programme framework (Quotes FP1, DOTS2, MO2).

It will be good if refresher training is given because sometimes there is an issue with the patient and we get confused so we go downstairs to the doctor so if training is given it will be good so that if we do not understand anything or there is something new it will get clear to us.

(DOTS Strategy Facilitator DOTS2, Primary level‐BMU)

So, further training should be done from time to time because a person should be upgraded along with his health. I think this is enough for now.

(District TB Focal Person FP1, Peshawar)

You get us trained a little. It will be good for us too. So, it happens that everyone comes to the same page. When a staff member and a medical officer give the same thing, then everyone will automatically come to the same page.

(Medical Officer MO2, Primary level‐BMU)

Patients became increasingly familiar with the care pathway over time. As implementation progressed, they described greater confidence in navigating counselling, referral and follow‐up procedures, suggesting that participation became easier with experience. However, practical barriers—including travel distance, financial constraints and medicine availability—continued to affect engagement throughout implementation.

### 3.6 4. Adoption

The theme emphasised the role of the senior managers and the willingness of the healthcare providers in successful implementation of the Opt‐TBD care package.

#### 3.6.1 Sub‐Theme 4A: Early Uptake By Bmus

Healthcare providers demonstrated strong willingness to adopt the Opt‐TBD care package within routine TB services. Despite initial concerns regarding increased workload from diabetes screening, counselling and follow‐up, the structured nature of the intervention enabled providers to rapidly incorporate these tasks into daily workflows. With increasing familiarity and practice, it became more organised and channelised, resulting in efficient and structured integration of diabetes management within routine TB care (Quotes DOTS1, FP1).

Burden on staff increases in terms of time and additional tasks of RBG and HbA1c and patients wait but it's manageable on a daily basis. The burden is placed, if there is a new patient, the registration will take at least 5 to 10 min. I must do RBG screening as per my role in the project which also takes time. So, there is an extra burden due to extra duties associated with the integrated care, but I will say it's not a problem for me. Additionally, I take blood samples of patients for HbA1c and send it to the nearby laboratory for a test. So, it's manageable and I manage it on a daily basis.

(DOTS Strategy Facilitator DOTS1, Tertiary level‐BMU).

In the beginning, there was a problem but now it is a regular routine. In the beginning, there was little problem in diagnosing (diabetes), then doing consultation and then to send him to the lab of tertiary care and reports, and then to communicate further. But now it is channelised, in the BMUs, it will be easily used in the routine.

(District TB Focal Person FP1, Peshawar)

We have a lot of patients on Monday, Tuesday, and Wednesday. We have a problem in these days. Patients have high flow and OPD is higher. So, it takes a little extra effort because we have an OPD to run… listening to them and counseling them takes extra time.

(District TB Focal Person FP1, Peshawar)

#### 3.6.2 Sub‐Theme 4B: Influence of Leadership and Programme Support

Active leadership and strategic direction from TB Control Programme managers significantly facilitated adoption of the integrated care package. Health professionals viewed guidance issued through the NTP as an important mechanism for introducing and embedding new practices within routine TB services. Participants explained that incorporating the integrated care package into official NTP guidance and programme guidelines would facilitate its integration into routine practice, encourage health professionals to implement the recommended activities consistently, and support wider adoption across facilities.

Regular programme meetings and ongoing managerial guidance fostered sustained provider engagement, accountability and motivation. Embedding diabetes care discussions into routine TB meetings ensured consistent implementation of best practices for TB–diabetes management, strengthening the overall delivery of integrated care. Overall, leadership was perceived as providing the organisational direction and support needed to facilitate the implementation and routine integration of the TB–diabetes care package.

Beyond strategic direction, participants emphasised that effective implementation also depended on organisational support through staff capacity building and workshops to improve understanding and acceptance of the integrated care package. They further suggested that clearly assigning responsibilities and establishing accountability would improve role clarity, minimise confusion regarding implementation responsibilities, and facilitate coordination of different components of the intervention. Health professionals perceived leadership as extending beyond issuing programme directives to providing the organisational support, programme guidance and clearly defined responsibilities needed to facilitate the implementation of integrated TB–diabetes care within routine practice.

Upon receiving a mission or direction, consistently follow and continue to implement the guidance from NTP as needed.

(Medical Officer MO2, Primary level‐BMU)

It is obvious that when we get a mission or a direction, then we will bond with it. Then it will be in our guidelines at every place. And then we follow it. There are still a lot of things that NTP keeps telling us from time to time, so we try to implement it.

(Medical Officer MO2, Primary level‐BMU)

Patient adoption evolved beyond initial participation. Early accounts focused on accepting diabetes screening, attending follow‐up appointments and beginning recommended lifestyle changes. With continued exposure to the intervention, these behaviours became integrated into participants’ daily routines, with patients describing sustained medication adherence, dietary modification and regular physical activity. However, difficulties accessing diabetes medicines and achieving glycaemic control limited full adoption for some participants.

I took my prescribed medication on time and followed a restricted diet, I did exercise.

(Patient P2, 55 years, Female)

### 3.7 5. Penetration

The theme evaluated the extent of Opt‐TBD care package amalgamation into routine TB services and highlights the strengths and issues that emerged as a result of collaboration within the care model.

#### 3.7.1 Sub‐Theme 5A: Degree of Integration into Daily Routines

Healthcare providers indicated that diabetes screening and counselling had become firmly integrated into routine TB services, supported by revised TB‐03 cards and TB‐01 registers that included dedicated sections for diabetes management. As providers became increasingly familiar with the integrated care package, they extended diabetes screening to extrapulmonary TB patients, beyond the original target group. This adaptation was reflected as a deliberate response to perceived clinical need, with providers recognising the importance of diabetes screening for all TB patients encountered in routine practice, suggesting that, once integrated into everyday service delivery, the intervention was viewed as sufficiently valuable to encourage providers to apply its principles beyond the original study population. Many advocated for the care model's continued implementation and expansion to other BMUs, viewing it as crucial to addressing the diabetes burden among TB patients (Quotes FP1, MO1, DOTS2).

As a general health worker, I can help the patients with diabetes and treat both diseases and patients and if the same patient can get help in their family and society then why not, I think it is a matter of honour for us and I would like that this should be started in other BMUs and further training and sessions should be done. I think this is a very good thing and we should do it.

(District TB‐Focal Person FP1, Peshawar)

The programme is extremely important due to increasing TB prevalence and diabetes among TB patients. It is ready for full implementation, making TB and diabetes management easier and benefiting patients, as previously suggested.

(Medical Officer MO1, Secondary level‐BMU)

In our routine care this change has taken place, who are not recruited in your project the extra pulmonary so we do their counselling as well and ask them about their diabetes, we ask whether any one at home have diabetes, or do you have such symptoms or any of your parents have diabetes, or do you get thirsty a lot or frequently experience urination at night, so this is one thing for us, that we have included this in the TB counselling. It is easy; there is no problem for us. There were some issues in the beginning, because we would forget certain things but now gratefully counselling and everything is done.

(DOTS Strategy Facilitator DOTS2, Primary level‐BMU)

#### 3.7.2 Sub‐Theme 5B: Collaboration With Specialised Care

The establishment of structured referral pathways to diabetes specialists enhanced collaboration between TB staff and specialised care providers (endocrinologists and diabetologists). This facilitated timely specialist intervention, structured follow‐up, and guidance on medication adherence and glycaemic control, improving continuity of care. However, challenges such as delayed HbA1c results persisted. These issues were gradually mitigated through improved coordination with laboratory personnel and the introduction of secure digital messaging, adhering to data protection standards (Quotes DOTS1).

In case of uncontrolled sugar, they are referred to an endocrinologist, a medical specialist, or a tertiary care hospital. They guide them further. During follow ups, feedback is taken from the patients about their sugar and medicines. A very good personal experience with the program.

(DOTS Strategy Facilitator DOTS1, Tertiary level‐BMU)

We have a little problem in the laboratory because it takes 3‐4 h in processing. So, if our liaison is formed with the laboratory, they do the HbA1c when they receive it, they do it early so the patient would not have to wait for long and don't come again I used to do it like this that I used to write the number of the patient and the share the report on the WhatsApp. So, then for the physicians they used to take printouts. I did not call the patient again. I used to send the report on WhatsApp.

(DOTS Strategy Facilitator DOTS1, Tertiary level‐BMU)

Penetration of the Opt‐TBD care package increased as diabetes‐related services became progressively embedded within routine TB care from the patients' perspective. Initially, patients primarily described diabetes screening, counselling, referral, medicines and provider support as additional services received alongside TB treatment. By Month 6, these components were increasingly perceived as routine and coordinated elements of their TB care pathway, with ongoing monitoring, counselling and specialist referral becoming expected aspects of their regular TB visits. This suggested greater integration of diabetes care within established TB service delivery pathways. Nevertheless, persistent challenges related to waiting times and access to diabetes medicines indicated that further strengthening of diabetes‐related resources is required to achieve full integration of the intervention into routine care (Quote P8, P6).

The medications (sugar) are expensive, which can be a burden, especially during times of economic inflation. While the program helps those with financial hardships, patients still have to pay for medications, adding to their financial strain.

(Patient P8, 52 years, Male)

Overall, everything was good quality‐wise, I had no problem except that the waiting part was a bit of an issue, thankfully Alhamdulillah everything was perfect.

(Patient P6, 32 years, Female)

### 3.8 6. Sustainability

The theme highlighted the critical factors for sustaining the care model, including financial support, intrinsic motivation and managerial engagement.

#### 3.8.1 Sub‐Theme 6A: Financial Incentives and Continuity

Healthcare providers emphasised that, although financial incentives aided service delivery, intrinsic motivation and recognition of patient benefits drove their continued commitment to the Opt‐TBD care package. Providers reported the structured care model had become embedded within their routine practice, independent of financial incentives. Nonetheless, patients highly valued travel reimbursements, especially those from remote or financially disadvantaged backgrounds, highlighting the necessity for ongoing financial support mechanisms. Providers, especially DOTS Strategy Facilitators, expressed concerns regarding long‐term financial sustainability due to dependence on external donor funding, underscoring the need for strategic policy engagement to secure future resources (Quotes DOTS2, FP1, P4, DOTS1).

The incentives you are giving us that is also possible. But we do the work anyway. So, one positive thing is that a person is encouraged with incentives. Like with the District TB Assistant, there were some problems but now it has been resolved. So, it makes the delivery process easier.

(DOTS Strategy Facilitator DOTS2, Primary level‐BMU)

So, personally, our (name of doctor) and DTO are committed. If the project is done or not, we will keep consulting the patient about TB and diabetes. If the patient is not cured, we will keep on guiding him. If the patient is sick and diagnosed with TB, we will give him some lifestyle modification and counselling.

(District TB Focal Person FP1, Peshawar)

I think financial assistance of the healthcare staff is not a hindrance.

(District TB Focal Person FP1, Peshawar)

I also remember that each time I visited, you provided money to cover the cost of traveling to the TB centre, which was a huge help. This level of support, both financially and medically, makes it much easier for patients like me to adhere to the treatment and get better. All these components—the free testing, the follow‐ups, and the travel assistance—have made a big difference in my recovery and are, in my view, the strongest parts of the care package.

(Patient P4, 45 years, Male)

We will have to see what is going on in the procurement or what does the government do because all the medicines of our TB are coming from outside, it is coming from (name of fund). We do not have any kind of resources from the national government or the provincial government. Everything is done from outside (abroad). So, this is the situation. If the government takes it up with the professional people's support, if they get some benefit, if they get some package, then that would be a benefit for them. It will be a source of attraction. Otherwise, it is very difficult for our government to take it up.

(DOTS Strategy Facilitator DOTS1, Tertiary level‐BMU)

#### 3.8.2 Sub‐Theme 6B: Long‐Term Embedding in Tb Programme

The TB Control Programme's ongoing commitment to improved patient outcomes provided a strong foundation for permanently embedding diabetes screening and management into routine care. Providers recommended national‐level integration of the Opt‐TBD model, supported by regular policy meetings and continued managerial engagement. Expanding and evaluating the care package across additional healthcare facilities, diagnostic centres and hospitals was suggested to standardise and sustain comprehensive TB–diabetes management (Quotes DOTS3, DOTS1, DOTS2, MO2).

This is an extremely important programme that needs to be implemented for a longer period of time. This is due to the fact that TB is becoming more prevalent every day, and diabetes is also extremely prevalent among those who have TB. It is ready for full implementation and nothing is missing. I have provided some suggestions in the past.

(DOTS Strategy Facilitator DOTS3, Secondary level‐BMU)

If it (care package) is merged into the TB Control Program, then it will be beneficial.

(DOTS Strategy Facilitator DOTS1, Tertiary level‐BMU)

The NTP (National TB Control Programme) people take it (care package) seriously. And I hope that God willing, they will take it seriously in the future.

(Medical Officer MO2, Primary level‐BMU)

It's a good thing If you implement this with us, like it is done in our facility, and in other hospitals, and diagnostic centres, it will be a good thing; this will facilitate the patient even more. He will have awareness; he will change his lifestyle. He will take care of other people as well. Like, he will stop smoking and engage in other activities. He will change his lifestyle and reduce his diabetes.

(DOTS Strategy Facilitator DOTS2, Primary level‐BMU)

### 3.9 Differences Across Patient Groups

While overall experiences were consistent across participants, gendered differences were observed. Female patients often faced additional social and logistical barriers—such as lack of accompaniment to health facilities, family responsibilities, and difficulty seeking care without male support. They also cited domestic challenges (e.g., caring for children or husbands) that affected their ability to adhere to follow‐ups. Male patients primarily reported financial concerns, including job loss or wage disruptions due to repeated facility visits. These findings underscore the need for gender‐sensitive support mechanisms in future scale‐up efforts.

### 3.10 Key Challenges and Enablers for Scaling Up the Opt‐TBD Care Model

This qualitative assessment demonstrated substantial implementation potential for the Opt‐TBD model to effectively integrate diabetes care within routine TB services. However, key barriers affecting the potential included delays in HbA1c testing, limited private spaces for counselling, inefficient referral mechanisms and uncertainties around financial sustainability due to the TB programme's reliance on external donor funding.

Conversely, several enabling factors enhanced the model's implementation potential. These included the co‐location of TB and diabetes services, provision of free diagnostic tests, structured referral systems, patient travel reimbursements and strong commitment from healthcare providers. Integration of the model within existing TB control infrastructure further supports its potential scalability at the national level.

## 4 Discussion

This study explored the implementation potential of integrating diabetes care into routine TB services in Pakistan, through the Opt‐TBD care package and contextually tailored implementation strategies. Patients and healthcare providers generally perceived the package as acceptable and appropriate, and providers described incorporating diabetes screening, counselling and referral activities within routine TB services. Implementation was supported by structured staff training, availability of essential resources, referral mechanisms and programme leadership. However, laboratory delays, privacy concerns, increased workload, referral challenges and dependence on external financial support remained important constraints that need consideration before wider implementation.

The high acceptability of integrated care observed across both time points in this study aligns with global evidence indicating improved patient engagement and adherence in similar TB–diabetes models [12, 27, 28]. A mixed‐methods study in Delhi, India found both qualitative and quantitative evidence for acceptability and feasibility of embedding NCDs and risk factors for NCDs screening within routine TB care at the DOTS centres [29]. A qualitative study of TB–diabetes screening in private primary care clinics in Yogyakarta, Indonesia, found that most clinics considered implementation of screening both acceptable and practical, with the majority of diabetes patients accepting the integrated screening [30]. A cross‐sectional study and a policy document review investigating the perceived acceptability and feasibility of integrating TB‐DM care reported high endorsement by the Malawian health workers [31]. In our study, patients valued integrated services provided at a single facility and financial incentives, particularly travel reimbursements. Nevertheless, delays associated with HbA1c testing emerged as a critical barrier affecting patients' daily wages, highlighting the need for rapid, point‐of‐care diagnostics—a challenge similarly noted in integrated care studies in India and Tanzania [28, 31]. Our findings show that acceptability is not just an initial reaction to a new health service but a judgement that persists and strengthens as patients accumulate the lived experience of the integrated care.

The appropriateness of integrating diabetes management within existing TB care structures was reinforced by streamlined workflows, allowing TB staff to incorporate diabetes services without major disruptions. Culturally tailored lifestyle counselling was particularly valued by patients, promoting informed lifestyle decisions. A qualitative study in Urban Nepal took a similar approach to ours, adapting generic WHO dietary advice in the context of socio‐cultural variation for people with diabetes, rather than introducing an external standard [32]. Another qualitative study of dietary management in South Africa concluded that diabetes interventions need to be tailored to local food environments [33]. An exploratory qualitative study of healthcare workers' perspectives in Ghana on barriers and facilitators to bidirectional screening of TB‐DM and a pilot feasibility study with mixed methods evaluation of integrating management of TB and diabetes in rural India incorporated culturally relevant education and counselling as an essential part of routine screening. The Ghana study qualitatively explored provider perspectives on barriers and facilitators to bidirectional TB–diabetes screening, while the rural India mixed‐methods feasibility study examined acceptability and feasibility of integrated TB–diabetes management [27, 34]. Both highlight the importance of adapting integrated care to local service and patient contexts. Our findings indicate appropriateness was about whether counselling was delivered in a way patients could fit it into their daily routines and existing food options. This suggests that perceived appropriateness may depend not only on service configuration but also on whether recommendations can be accommodated within patients' everyday routines and available food choices. However, the increased patient load at some facilities indicated a potential need for additional staffing or workflow adjustments, a barrier similarly reported in TB‐NCD integration efforts in Ethiopia, Peru and Nigeria [35, 36, 37, 38].

Feasibility was supported by consistent resource availability, including diagnostic tools (glucometers, HbA1c kits) and financial incentives, supporting delivery of the integrated care package. Yet, sustainable feasibility depends on consistent financing and strategic policy‐level support, as reliance on external funding poses long‐term risks. Providers stressed the necessity of securing local procurement of essential supplies, echoing concerns from Malawi and China where inconsistent funding hindered programme sustainability [31, 39, 40, 41]. While structured referrals to diabetes specialists improved feasibility, logistical challenges related to blood sample transportation and patient navigation highlighted the importance of robust communication channels, an issue previously identified in South African chronic disease care models [39, 42].

Adoption was reflected in providers’ accounts of incorporating diabetes screening, counselling and referral activities into routine TB care. This is consistent with the bidirectional TB–diabetes screening study in Mexico, where healthcare workers incorporated integrated activities into routine service delivery [43]. The scale‐up of diabetes screening to extrapulmonary TB patients by the DOTS *Strategy Facilitators* beyond the study population is a notable finding relative to the comparison literature, most of which reports on adherence to or deviation from a protocol rather than provider‐initiated expansion. This finding is consistent with observations from implementation science literature that NCD programme delivery works better when the community health workers understand the reason behind each individual task instead of robotically following a fixed protocol; a scoping review on the role of community health workers in NCD prevention showed that sustainable integration depends on adapting the additional tasks into routine care rather than treating it as an additional burdensome task [44]. Our data indicate that this adaptation process has already been initiated in the Opt‐TBD care package, even before any policy change, a more advanced stage of integration than acceptability or feasibility‐only outcomes reported in Delhi, Indonesia, Mexico and Malawian studies cited above, none of which report on voluntarily expanding beyond the target population.

The extension of screening beyond the original target population may be understood as a form of local adaptation or reinvention, consistent with Rogers' diffusion‐of‐innovation theory, whereby users modify an innovation while retaining its core purpose [45]. From an implementation perspective, this finding also illustrates the potential tension between adaptation and fidelity [46]. Provider‐led adaptation may indicate emerging ownership and routinisation of integrated care; however, expansion beyond the defined intervention population may also have implications for fidelity, clinical appropriateness and resource requirements. These implications should therefore be evaluated during wider implementation rather than assuming that such adaptation necessarily represents greater adoption or sustainability.

Training emerged as a critical facilitator of successful adoption. Providers recommended periodic refresher courses to maintain best practices, aligning with global evidence highlighting significant gaps in training for TB–diabetes integration. Systematic and scoping reviews found that inadequate training was a major barrier to integrated care in LMICs, leading to suboptimal practices [37, 47]. Qualitative research from Ethiopia further reinforced that insufficient diabetes management training posed considerable challenges to integrated service delivery [48]. This is in line with our study findings that healthcare providers need structured training to screen and counsel for diabetes confidently. In Ethiopia, this knowledge gap was identified as an unresolved barrier to integration, whereas in our study, the refresher training appears to have addressed it to some extent. Thus, structured training models, such as the training‐of‐trainers approach utilised in this study, remain essential for successful and sustainable implementation. Within participating BMUs, penetration was reflected in the incorporation of diabetes‐related activities into routine processes, including modified TB registers and strengthened linkages with diabetes specialists. Laboratory delays and inter‐facility communication gaps nevertheless highlighted the need to strengthen communication and information‐sharing systems, potentially including appropriate digital approaches [49, 50, 51, 52, 53, 54].

A central systems‐level challenge was the dependence of free integrated service delivery on external financing. Ensuring long‐term sustainability requires embedding the Opt‐TBD model within national TB strategies, securing financial resources, and continuous policy engagement. While healthcare providers demonstrated intrinsic motivation to sustain integrated care, financial support mechanisms were crucial, mirroring experiences from integrated programmes in Nigeria and Bangladesh, where national health financing schemes enhanced long‐term viability [36, 37, 55]. Regular policy dialogues with TB control programmes were also emphasised as essential for institutionalising integrated care pathways [56]. These findings are consistent with broader international policy priorities supporting integrated TB‐NCD care, including WHO's End TB Strategy, the Union's Collaborative Framework for TB and Diabetes, and Sustainable Development Goals (SDG 3.4) [57, 58]. The WHO Pakistan Country Cooperation Strategy also supports integrated disease management approaches, underscoring the study's relevance [59, 60]. However, the present findings should be interpreted as formative evidence on implementation potential.

Taken together, our findings suggest that implementation potential depended not on any single intervention component, but on the interaction between perceived value to patients and providers, fit with routine TB workflows, staff capability, functioning referral and diagnostic pathways, and the availability of sustained programme resources.

A key strength of this study is its real‐world implementation within routine TB care facilities, allowing practical assessment of the intervention's implementation potential in diverse healthcare settings (primary, secondary and tertiary). Including patients and healthcare providers from varied facility levels offered a rich, multi‐perspective analysis, effectively capturing context‐specific barriers and facilitators critical for successful scale‐up. Furthermore, while most feasibility studies focus narrowly on feasibility and acceptability, this study deliberately assessed the implementation potential using a broader range of implementation outcomes to inform practical decisions around refinement and wider implementation of the integrated TB and diabetes care. By integrating two complementary frameworks, we clearly distinguished between the operational (how‐to) aspects of implementation (guided by CFIR's Process domain) and the evaluative criteria or outcomes of successful implementation (as captured by Proctor's outcomes). This integration strengthened methodological rigour and provided precise insights into both the intervention's operational feasibility and its implementation outcomes.

### 4.1 Limitations

This study has several limitations. First, it was conducted in selected TB facilities in two provinces of Pakistan, which may limit the transferability of findings to settings with different healthcare infrastructures, socioeconomic conditions and cultural contexts. The patient sample also had limited sociodemographic diversity, as most participants had no formal education, all were married, and the majority lived in urban areas. Recruitment varied across facility levels, with fewer participants recruited from the tertiary‐level facility, which may have constrained comparisons across settings. Second, patients and healthcare providers were interviewed at the facilities where they received care or worked, creating a potential risk of socially desirable responses. Although interviews were conducted privately and confidentiality was assured, some participants may still have been reluctant to express critical views. The gender and positionality of the research assistants may also have influenced participant disclosure and interpretation of the findings. In addition, interviews were conducted in participants' preferred local languages and translated into English, which may have resulted in some loss of cultural or contextual meaning. Third, the study focused primarily on the early implementation period between Months 3 and 6 and on short‐term feasibility and implementation outcomes. Longer‐term clinical outcomes, sustained integration into routine services, and the durability of implementation after external financial, organisational and research support is reduced therefore remain uncertain. Identified operational challenges, including laboratory delays and referral inefficiencies, also require further examination and optimisation in subsequent implementation research. Finally, although the qualitative findings provided rich insights into implementation experiences, incorporating longitudinal quantitative clinical outcomes, such as HbA1c trends and other measures of clinical effectiveness, would enable a more comprehensive assessment of the intervention's effectiveness and long‐term sustainability.

## 5 Conclusion

Our study indicates promising implementation potential for integrating the Opt‐TBD care package within routine TB services, while identifying important barriers that require attention before wider‐scale implementation. The Opt‐TBD care package integration progressed further than acceptability alone, as DOTS Strategy Facilitators independently screened extrapulmonary TB patients outside the study cohort (pulmonary TB cases). Without any requirement to do so, suggesting that staff had internalised the rationale for integrated care rather than following a fixed task; a strong indicator of adoption. By aligning with international frameworks such as the WHO's End TB Strategy and The Union's Collaborative Framework for TB and Diabetes, as well as national health priorities, the Opt‐TBD package, with targeted efforts to address identified barriers, offers a scalable, contextually relevant model for addressing TB–diabetes multimorbidity in resource‐limited settings.

However, three addressable barriers will determine whether this adoption is sustainable at scale. First, delayed HbA1c results indicate a need for point‐of‐care HbA1c testing at each level rather than continued reliance on centralised laboratory processing. Second, patients with low literacy levels struggled to navigate referrals from primary level to the specialist at tertiary hospitals, requiring ward‐based specific referral pathways as proposed by the healthcare providers themselves. Finally, providers acknowledged that current diabetes care depends on donor funding, and long‐term continuity cannot be assured by staff motivation alone. There is a need for embedding the Opt‐TBD care package into the DOTS Strategy Facilitators training curriculum and cost budget line within national TB programme planning. Our findings suggest that the sustainability risk of the Opt‐TBD care package is concentrated in referral clarity, diagnostic turnaround and allocated financing rather than provider adoption alone, a distinction that should help inform the design of any provincial and national scale‐up planning.

## Author Contributions

**Saima Afaq:** conceptualisation, funding acquisition, writing – original draft, writing – review and editing, resources, visualisation, supervision, investigation, validation. **Syeda Fatima Jamal:** project administration, formal analysis, writing – review and editing, data curation, visualisation. **Aliya Rehman:** visualisation, formal analysis, data curation, writing – review and editing. **Urooj Ashfaq:** data curation, formal analysis, writing – review and editing. **Zala:** data curation, visualization, writing – review and editing, formal analysis, project administration. **Muhammad Asim:** data curation, formal analysis, writing – review and editing. **Farman Ullah Khan:** data curation. **Suneel Gill:** data curation. **Haroon Latif Khan:** conceptualisation, supervision. **Mariyam Sarfraz:** conceptualisation, methodology, writing – review and editing. **Zohaib Khan:** conceptualisation, supervision. **Kamran Siddiqi:** conceptualisation, supervision, writing – review and editing. **Hannah Jennings:** writing – review and editing.

## Ethics Statement

Ethics approval for this study has been obtained from the National Bioethics Committee of Pakistan (Reference No: NBCR‐1010). Written informed consent was taken from all study participants.

## Consent

Written consent for publication was taken from all participants involved in the study.

## Conflicts of Interest

The authors declare no conflicts of interest.

## Data Availability

The data sets used and/or analysed during the current study are available from the corresponding author upon reasonable request.

## References

[1] M. D. Fenta , O. A. Ogundijo , A. A. A. Warsame , and A. G. Belay , “Facilitators and Barriers to Tuberculosis Active Case Findings in Low‐ and Middle‐Income Countries: A Systematic Review of Qualitative Research,” BMC Infectious Diseases 23, no. 1 (2023): 515, 10.1186/s12879-023-08502-7. PMC10405492 37550614

[2] Ministry of National Health Services, Regulations and Coordination , “National TB Control Programme,” 2021.

[3] CDC , “Tuberculosis Risk Factors, Tuberculosis (TB),” February 5, 2025, https://www.cdc.gov/tb/risk-factors/index.html.

[4] C. Y. Jeon and M. B. Murray , “Diabetes Mellitus Increases the Risk of Active Tuberculosis: A Systematic Review of 13 Observational Studies,” PLoS Medicine 5, no. 7 (2008): e152, 10.1371/journal.pmed.0050152. PMC2459204 18630984

[5] R. H. Al‐Rifai , F. Pearson , J. A. Critchley , and L. J. Abu‐Raddad , “Association Between Diabetes Mellitus and Active Tuberculosis: A Systematic Review and Meta‐Analysis,” PLoS One 12, no. 11 (2017): e0187967, 10.1371/journal.pone.0187967. PMC5697825 29161276

[6] M. A. Baker , A. D. Harries , C. Y. Jeon , et al., “The Impact of Diabetes on Tuberculosis Treatment Outcomes: A Systematic Review,” BMC Medicine 9, no. 1 (2011): 81, 10.1186/1741-7015-9-81. PMC3155828 21722362

[7] S. Gautam , N. Shrestha , S. Mahato , T. P. A. Nguyen , S. R. Mishra , and G. Berg‐Beckhoff , “Diabetes Among Tuberculosis Patients and Its Impact on Tuberculosis Treatment in South Asia: A Systematic Review and Meta‐Analysis,” Scientific Reports 11, no. 1 (2021): 2113, 10.1038/s41598-021-81057-2. PMC7822911 33483542

[8] S. S. Habib , S. Rafiq , W. Z. Jamal , et al., “Engagement of Private Healthcare Providers for Case Finding of Tuberculosis and Diabetes Mellitus in Pakistan,” BMC Health Services Research 20, no. 1 (2020): 328, 10.1186/s12913-020-05217-2. PMC7168982 32306961

[9] A. A. Boadu , M. Yeboah‐Manu , S. Osei‐Wusu , and D. Yeboah‐Manu , “Tuberculosis and Diabetes Mellitus: The Complexity of the Comorbid Interactions,” International Journal of Infectious Diseases 146 (2024): 107140, 10.1016/j.ijid.2024.107140. 38885832

[10] M. Khattak , A. Rehman , T. Muqaddas , et al., “Tuberculosis (TB) Treatment Challenges in TB‐Diabetes Comorbid Patients: A Systematic Review and Meta‐Analysis,” Annals of Medicine 56, no. 1 (2024): 2313683, 10.1080/07853890.2024.2313683. PMC10863515 38346381

[11] M. D. Ngo , S. Bartlett , and K. Ronacher , “Diabetes‐Associated Susceptibility to Tuberculosis: Contribution of Hyperglycemia vs. Dyslipidemia,” Microorganisms 9, no. 11 (2021): 2282, 10.3390/microorganisms9112282. PMC8620310 34835407

[12] R. van Crevel and J. A. Critchley , “The Interaction of Diabetes and Tuberculosis: Translating Research to Policy and Practice,” Tropical Medicine and Infectious Disease 6, no. 1 (2021): 8, 10.3390/tropicalmed6010008. PMC7838867 33435609

[13] U. Abbas , K. I. Masood , A. Khan , et al., “Tuberculosis and Diabetes Mellitus: Relating Immune Impact of Co‐Morbidity With Challenges in Disease Management in High Burden Countries,” Journal of Clinical Tuberculosis and Other Mycobacterial Diseases 29 (2022): 100343, 10.1016/j.jctube.2022.100343. PMC9720570 36478777

[14] D. M. Milice , I. Macicame , and J. L Peñalvo , “The Collaborative Framework for the Management of Tuberculosis and Type 2 Diabetes Syndemic in Low‐ and Middle‐Income Countries: A Rapid Review,” BMC Public Health 24, no. 1 (2024): 738, 10.1186/s12889-024-18256-9. PMC10921776 38454428

[15] S. Afaq , Z. Zala , S. Aleem , et al., “Implementation Strategies for Providing Optimised Tuberculosis and Diabetes Integrated Care in LMICs (POTENTIAL): Protocol for a Multiphase Sequential and Concurrent Mixed‐Methods Study,” BMJ Open 14, no. 11 (2024): e093747, 10.1136/bmjopen-2024-093747. PMC11603734 39609009

[16] S. Aleem , Z. Khan , B. A. Khan , P. Zala , and S. Afaq , “Readiness Assessment as a Strategy, Not an Afterthought: Leveraging Implementation Science to Integrate Tuberculosis Comorbidity Care in Pakistan's Health System,” Frontiers in Tuberculosis 4 (2026): 1809118, 10.3389/ftubr.2026.1809118. PMC13494971 42638741

[17] E. K. Proctor , B. J. Powell , and J. C. McMillen , “Implementation Strategies: Recommendations for Specifying and Reporting,” Implementation Science 8, no. 1 (2013): 139, 10.1186/1748-5908-8-139. PMC3882890 24289295

[18] B. Saunders , N. E. Foster , J. C. Hill , et al., “First Contact Practitioners' (FCPs) and General Practitioners' Perceptions Towards FCPs Delivering Vocational Advice to Patients With Musculoskeletal Conditions: A Qualitative Investigation of the Implementation Potential of the I‐Swap Initiative,” Journal of Occupational Rehabilitation 32, no. 1 (2022): 147–155, 10.1007/s10926-021-09992-5. PMC8858917 34241768

[19] G. M. Curran , M. Bauer , B. Mittman , J. M. Pyne , and C. Stetler , “Effectiveness‐Implementation Hybrid Designs: Combining Elements of Clinical Effectiveness and Implementation Research to Enhance Public Health Impact,” Medical Care 50, no. 3 (2012): 217–226, 10.1097/MLR.0b013e3182408812. PMC3731143 22310560

[20] A. R. Elwy , A. D. Wasan , A. G. Gillman , et al., “Using Formative Evaluation Methods to Improve Clinical Implementation Efforts: Description and an Example,” Psychiatry Research 283 (2020): 112532, 10.1016/j.psychres.2019.112532. 31477261

[21] H. Pinnock , M. Barwick , C. R. Carpenter , et al., “Standards for Reporting Implementation Studies (StaRI) Statement,” BMJ 356 (2017): i6795, 10.1136/bmj.i6795. PMC5421438 28264797

[22] J. S. Holtrop , P. A. Estabrooks , B. Gaglio , et al., “Understanding and Applying the RE‐AIM Framework: Clarifications and Resources,” Journal of Clinical and Translational Science 5, no. 1 (2021): e126, 10.1017/cts.2021.789. PMC8327549 34367671

[23] M. U. Haq , S. G. Hinderaker , R. Fatima , et al., “Are Patients With Pulmonary Tuberculosis Identified by Active and by Passive Case Detection Different? A Cross‐Sectional Study in Pakistan,” International Journal of Infectious Diseases 121 (2022): 39–46, 10.1016/j.ijid.2022.04.055. 35489633

[24] S. Aleem , S. Afaq , B. Ahmad , Zala , H. Latif Khan , and Z. Khan , “From Silos to Synergy: Assessing Tuberculosis Basic Management Units Readiness for Integrated Diabetes Mellitus Care Across Different Districts of Pakistan,” Primary Health Care Research & Development 27 (2026): e47, 10.1017/S146342362610111X. PMC13080535 41958323

[25] N. K. Gale , G. Heath , E. Cameron , S. Rashid , and S. Redwood , “Using the Framework Method for the Analysis of Qualitative Data in Multi‐Disciplinary Health Research,” BMC Medical Research Methodology 13, no. 1 (2013): 117, 10.1186/1471-2288-13-117. PMC3848812 24047204

[26] WageIndicator Foundation , “Minimum Wage Increased in Balochistan, Khyber Pakhtunkhwa and Punjab, Pakistan From 01 July 2023 ‐ September 02, 2023” (WageIndicator Foundation, 2026), https://wageindicator.org/work/minimum-wage/updates/2023/minimum-wage-increased-in-balochistan-khyber-pakhtunkhwa-and-punjab-pakistan-from-01-july-2023-september-02-2023/.

[27] R. Joshi , D. Behera , G. L. Di Tanna , M. A. Ameer , K. Yakubu , and D. Praveen , “Integrated Management of Diabetes and Tuberculosis in Rural India—Results From a Pilot Study,” Frontiers in Public Health 10 (2022): 766847, 10.3389/fpubh.2022.766847. PMC9127505 35619802

[28] J. L. Z. Nyirenda , A. Bockey , D. Wagner , and B. Lange , “Effect of Tuberculosis (TB) and Diabetes Mellitus (DM) Integrated Healthcare on Bidirectional Screening and Treatment Outcomes Among TB Patients and People Living With DM in Developing Countries: A Systematic Review,” Pathogens and Global Health 117, no. 1 (2023): 36–51, 10.1080/20477724.2022.2046967. PMC9848381 35296216

[29] T. Anand , J. Kishore , P. Isaakidis , et al., “Integrating Screening for Non‐Communicable Diseases and Their Risk Factors in Routine Tuberculosis Care in Delhi, India: A Mixed‐Methods Study,” PLoS One 13, no. 8 (2018): e0202256, 10.1371/journal.pone.0202256. PMC6107155 30138331

[30] D. A. Prakoso , W. Istiono , Y. Mahendradhata , and M. Arini , “Acceptability and Feasibility of Tuberculosis‐Diabetes Mellitus Screening Implementation in Private Primary Care Clinics in Yogyakarta, Indonesia: A Qualitative Study,” BMC Public Health 23, no. 1 (2023): 1908, 10.1186/s12889-023-16840-z. PMC10546762 37789310

[31] J. L. Nyirenda , E. Mbemba , M. Chirwa , et al., “Acceptability and Feasibility of Tuberculosis and Diabetes Mellitus Bidirectional Screening and Joint Treatment Services in Malawi: A Cross‐Sectional Study and a Policy Document Review,” BMJ Open 13, no. 1 (2023): e062009, 10.1136/bmjopen-2022-062009. PMC9827251 36609325

[32] L. Caperon , A. Arjyal , P. K. C., et al., “Developing a Socio‐Ecological Model of Dietary Behaviour for People Living With Diabetes or High Blood Glucose Levels in Urban Nepal: A Qualitative Investigation,” PLoS One 14, no. 3 (2019): e0214142, 10.1371/journal.pone.0214142. PMC6433239 30908526

[33] N. Nkaule , N. V. Khosa , Z. Ncane , and S. C. Nomatshila , “Dietary Perceptions and Challenges in the Management of Type 2 Diabetes in Rural Communities of the Eastern Cape, South Africa: A Qualitative Study,” Frontiers in Clinical Diabetes and Healthcare 7 (2026): 1835644, 10.3389/fcdhc.2026.1835644. PMC13388071 42488689

[34] R. S. Salifu and K. W. Hlongwana , “Barriers and Facilitators to Bidirectional Screening of TB‐DM in Ghana: Healthcare Workers' Perspectives,” PLoS One 15, no. 7 (2020): e0235914, 10.1371/journal.pone.0235914. PMC7360027 32663233

[35] A. L. Byrne , B. J. Marais , C. D. Mitnick , et al., “Feasibility and Yield of Screening for Non‐Communicable Diseases Among Treated Tuberculosis Patients in Peru,” International Journal of Tuberculosis and Lung Disease 22, no. 1 (2018): 86–92, 10.5588/ijtld.17.0381. 29297431

[36] N. Ekeke , E. Aniwada , J. Chukwu , et al., “Screening Diabetes Mellitus Patients for Tuberculosis in Southern Nigeria: A Pilot Study,” Advances in Respiratory Medicine 88, no. 1 (2020): 6–12, 10.5603/ARM.2020.0072. 32153002

[37] C. D. Foo , P. Shrestha , L. Wang , et al., “Integrating Tuberculosis and Noncommunicable Diseases Care in Low‐ and Middle‐Income Countries (LMICs): A Systematic Review,” PLoS Medicine 19, no. 1 (2022): e1003899, 10.1371/journal.pmed.1003899. PMC8806070 35041654

[38] M. Nunemo , K. Gidebo , E. Woticha , and Y. K. Lemu , “Integration Challenges and Opportunity of Implementing Non‐Communicable Disease Screening Intervention With Tuberculosis Patient Care: A Mixed Implementation Study,” Risk Management and Healthcare Policy 16 (2023): 2609–2633, 10.2147/RMHP.S432943. PMC10693204 38045564

[39] G. Gwaza , A. Plüddemann , M. McCall , and C. Heneghan , “Integrated Diagnosis in Africa's Low‐ and Middle‐Income Countries: What Is It, What Works, and for Whom? A Realist Synthesis,” International Journal of Integrated Care 24, no. 3 (2024): 20, 10.5334/ijic.7788. PMC11396343 39280804

[40] Q. Long , W. X. Jiang , H. Zhang , J. Cheng , S. L. Tang , and W. B. Wang , “Multi‐Source Financing for Tuberculosis Treatment in China: Key Issues and Challenges,” Infectious Diseases of Poverty 10, no. 1 (2021): 17, 10.1186/s40249-021-00809-4. PMC7943260 33750460

[41] A. Abdullah , L. B. Rawal , S. Bhattarai , et al., “Integrated Care for Tuberculosis (TB) and Diabetes Mellitus (DM) Comorbidity in Asian Countries: Health System Challenges and Opportunities,” 2026, https://www.semanticscholar.org/paper/Integrated-care-for-tuberculosis-(TB)-and-diabetes-Abdullah-Rawal/142df788293ac5b6823145c1d722d31f302b1203.

[42] O. H. Mahomed , S. Asmall , and A. Voce , “Sustainability of the Integrated Chronic Disease Management Model at Primary Care Clinics in South Africa,” African Journal of Primary Health Care & Family Medicine 8, no. 1 (2016): e1–e7, 10.4102/phcfm.v8i1.1248. PMC5125260 28155314

[43] M. Castellanos‐Joya , G. Delgado‐Sánchez , L. Ferreyra‐Reyes , et al., “Results of the Implementation of a Pilot Model for the Bidirectional Screening and Joint Management of Patients With Pulmonary Tuberculosis and Diabetes Mellitus in Mexico,” PLoS One 9, no. 9 (2014): e106961, 10.1371/journal.pone.0106961. PMC4167852 25229236

[44] T. Haregu , P. Delobelle , A. Shrestha , et al., “Shifting Roles of Community Health Workers in the Prevention and Management of Noncommunicable Disease During the COVID‐19 Pandemic: A Scoping Review,” Health Policy and Planning 39, no. 7 (2024): 771–781, 10.1093/heapol/czae049. PMC11308610 38910332

[45] J. A. García‐Avilés , “Diffusion of Innovation,” in The International Encyclopedia of Media Psychology, ed. J. Van den Bulck (John Wiley & Sons, 2020), 1–8, 10.1002/9781119011071.iemp0137.

[46] S. W. Stirman , C. J. Miller , K. Toder , and A. Calloway , “Development of a Framework and Coding System for Modifications and Adaptations of Evidence‐Based Interventions,” Implementation Science 8, no. 1 (2013): 65, 10.1186/1748-5908-8-65. PMC3686699 23758995

[47] J. O'Donovan , C. O'Donovan , I. Kuhn , S. E. Sachs , and N. Winters , “Ongoing Training of Community Health Workers in Low‐Income Andmiddle‐Income Countries: A Systematic Scoping Review of the Literature,” BMJ Open 8, no. 4 (2018): e021467, 10.1136/bmjopen-2017-021467. PMC5931295 29705769

[48] M. H. Workneh , G. A. Bjune , and S. A. Yimer , “Assessment of Health System Challenges and Opportunities for Possible Integration of Diabetes Mellitus and Tuberculosis Services in South‐Eastern Amhara Region, Ethiopia: A Qualitative Study,” BMC Health Services Research 16, no. 1 (2016): 135, 10.1186/s12913-016-1378-6. PMC4837556 27095028

[49] A. Adekunle Oyeyemi Adeniyi , A. Jeremiah Olawumi Arowoogun , C. Rawlings Chidi , O. Chioma Anthonia Okolo , and B. Oloruntoba Babawarun , “The Impact of Electronic Health Records on Patient Care and Outcomes: A Comprehensive Review,” World Journal of Advanced Research and Reviews 21, no. 2 (2024): 1446–1455, 10.30574/wjarr.2024.21.2.0592.

[50] H. Elsey , Z. Al Azdi , S. Regmi , et al., “Scaling Up Tobacco Cessation Within TB Programmes: Findings From a Multi‐Country, Mixed‐Methods Implementation Study,” Health Research Policy and Systems 20, no. 1 (2022): 43, 10.1186/s12961-022-00842-1. PMC9014631 35436896

[51] X. Liu , P. Sutton , R. McKenna , et al., “Evaluation of Secure Messaging Applications for a Health Care System: A Case Study,” Applied Clinical Informatics 10, no. 1 (2019): 140–150, 10.1055/s-0039-1678607. PMC6393161 30812040

[52] H. Myburgh , R. P. H. Peters , T. Hurter , C. J. Grobbelaar , and G. Hoddinott , “Transition to an In‐Facility Electronic Tuberculosis Register: Lessons From a South African Pilot Project,” Southern African Journal of HIV Medicine 21, no. 1 (2020): 1025, 10.4102/sajhivmed.v21i1.1025. PMC7059247 32158556

[53] C. Robbiati , M. E. Tosti , G. Mezzabotta , et al., “Improving TB Surveillance and Patients' Quality of Care Through Improved Data Collection in Angola: Development of an Electronic Medical Record System in Two Health Facilities of Luanda,” Frontiers in Public Health 10 (2022): 745928, 10.3389/fpubh.2022.745928. PMC9009439 35433613

[54] S. Satyanarayana , A. M. V. Kumar , N. Wilson , A. Kapur , A. D. Harries , and R. Zachariah , “Taking on the Diabetes‐Tuberculosis Epidemic in India: Paving the Way Through Operational Research,” Public Health Action 3, no. Suppl 1 (2013): S1–S2, 10.5588/pha.13.0039. PMC4463137 26393061

[55] K. Islam , R. Huque , K. M. Saif‐Ur‐Rahman , A. N. M. Ehtesham Kabir , and A. H. M. Enayet Hussain , “Implementation Status of Non‐Communicable Disease Control Program at Primary Health Care Level in Bangladesh: Findings From a Qualitative Research,” Public Health in Practice (Oxford, England) 3 (2022): 100271, 10.1016/j.puhip.2022.100271. PMC9461504 36101774

[56] W. M. Khan , H. Smith , E. Qadeer , and S. Hassounah , “Knowledge and Perceptions of National and Provincial Tuberculosis Control Programme Managers in Pakistan About the WHO Stop TB Strategy: A Qualitative Study,” JRSM Open 8, no. 1 (2016): 2054270416675084, 10.1177/2054270416675084. PMC5298435 28203383

[57] “Noncommunicable Diseases,” accessed August 2, 2026, https://www.who.int/news-room/fact-sheets/detail/noncommunicable-diseases.

[58] “World Diabetes Day 2023: Equitable Access to Care for People With TB and Diabetes,” accessed August 2, 2026, https://www.who.int/news/item/13-11-2023-world-diabetes-day-2023-equitable-access-to-care-for-people-with-tb-and-diabetes.

[59] “Global Tuberculosis Report 2024,” accessed August 2, 2026, https://www.who.int/teams/global-tuberculosis-programme/tb-reports/global-tuberculosis-report-2024.

[60] “Governance of TB Programs,” accessed August 2, 2026, https://www.stoptb.org/resources/governance-tb-programs.

